# C-Terminal Analogues of Camostat Retain TMPRSS2 Protease Inhibition: New Synthetic Directions for Antiviral Repurposing of Guanidinium-Based Drugs in Respiratory Infections

**DOI:** 10.3390/ijms26146761

**Published:** 2025-07-15

**Authors:** Bill T. Ferrara, Elinor P. Thompson, Giovanni N. Roviello, Thomas F. Gale

**Affiliations:** 1 School of Science, Faculty of Engineering and Science, University of Greenwich, Central Avenue, Chatham Maritime, Kent ME4 4TB, UK; 2 CNR Institute of Biostructures and Bioimaging, Via Tommaso De Amicis 95, 80145 Naples, Italy

**Keywords:** COVID-19, antiviral therapy, drug repurposing, respiratory virus, SARS-CoV-2, camostat, TMPRSS2, protease inhibitor

## Abstract

The recent global coronavirus pandemic highlighted the ever-present threat of respiratory virus outbreaks and the consequent need for ongoing research into antiviral therapy. To this end, structural analogues of the guanidinium-based drug camostat mesylate have been synthesised to probe their potential inhibition of Transmembrane Serine Protease 2 (TMPRSS2), a human protease that is essential for infection by many respiratory viruses, including Severe Acute Respiratory Syndrome Coronavirus 2 (SARS-CoV-2). Our in vitro fluorescence-based protease assays and supporting computational docking studies suggest that C-terminal camostat analogues retain TMPRSS2 inhibition potencies (IC_50_ = 1–3 nM, BE = −6.6 to −7.0 kcal/mol) that match or exceed that of the parent drug. Analogues **1c** and **1d** emerge as lead candidates in this regard, thereby validating the rationale behind C-terminal structural modifications and highlighting these derivatives as promising scaffolds for the future development of targeted antiviral therapeutics. Replacement of camostat’s ester functionality with peptide linkages largely preserves non-covalent binding but disrupts in vitro protease inhibition, findings consistent with the parent drug’s known role as an acylating suicide inhibitor. Docking studies confirm that the replacement of aromatic residues with flexible, equivalent-length alkyl chains is detrimental to drug binding. These function and binding data offer new directions for the synthesis of further analogues of camostat and of other guanidinium-based protease inhibitors that have yet to be refined via structure–activity relationship studies. Further investigation will support tailoring this class of drugs for repurposing in antiviral therapy.

## 1. Introduction

The persistent threat of viral respiratory diseases highlights the critical need for ongoing research into small-molecule antiviral drugs. Although the acute phase of the COVID-19 pandemic has passed [1], the potential for future coronavirus outbreaks necessitates the exploration of innovative therapeutic strategies. Drug repurposing offers a promising approach to address this challenge, accelerating the development of effective treatments by taking advantage of the benign safety profiles of already-approved drugs [2]. Recent studies have focused on developing or repositioning small-molecule drugs to inhibit TMPRSS2, a trypsin-like human serine protease that is hijacked by SARS-CoV-2 [3,4], influenza A [5], metapneumovirus [6] and other respiratory viruses [7,8] for eukaryotic cell entry and consequent viral infection.

**Figure 1:**
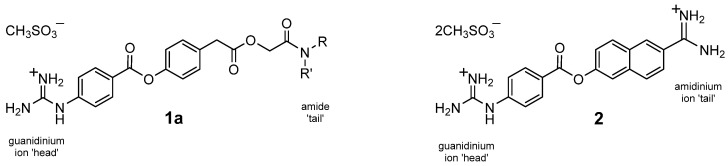
The molecular structures of camostat mesylate (**1a**) and nafamostat mesylate (**2**).

The physiological functions of TMPRSS2 remain incompletely defined, and evidence from proven inhibitors suggests that the disruption of this protease may incur only minor side effects. For example, MM3122, a potent and selective ketobenzothiazole peptidomimetic, reversibly covalent inhibitor of TMPRSS2, is a lead antiviral candidate for clinical development, which exhibits metabolic stability and a favourable safety profile in mouse models [9]. Camostat mesylate (**1a**, Figure 1; MW: 494.6 g/mol) and the structurally related drug nafamostat mesylate (**2**; MW: 539.6 g/mol) similarly disruptively bind TMPRSS2 within its catalytic cleft [4,10]. These synthetic guanidinium-functionalised drugs are already clinically approved in Japan and South Korea for a variety of roles in anticoagulant therapy and the treatment of pancreatitis pain and reflux esophagitis.

This established safety clearance opens up potential for their relatively rapid repurposing in antiviral therapy [11]. The further appeal of this strategy arises from the transmembrane location of the 492-residue TMPRSS2 and its abundance at the surface of human airway cells. As a consequence of the host cell-encoded enzyme function, antiviral activity demonstrated by novel camostat analogues may exhibit reduced susceptibility to resistance mechanisms, which typically arise rapidly when therapeutics target viral-encoded proteins. Although the COVID-19 clinical efficacy [12] and TMPRSS2 binding site mechanism [4,13] of camostat have been the focus of extensive investigation, synthetic structure–activity relationship (SAR) optimisation with a specific emphasis on antiviral activity remains largely unexplored [14]. Herein, we present the synthesis and assessment of camostat analogues, leveraging computational docking studies and fluorescence-based TMPRSS2 in vitro assays to suggest directions for future antiviral drug design. Given that most protease inhibitors exhibit varying degrees of activity across multiple targets, we propose that camostat and nafamostat can serve as lead scaffolds with scope for enhanced TMPRSS2-selective binding potency through strategic structural modifications—an avenue we explore in this study. Camostat mesylate has demonstrated therapeutic potential against SARS-CoV-2 in cell-based and tissue-based studies [15,16]. By disrupting TMPRSS2-mediated priming of the viral spike protein, camostat shuts down one of the two key membrane fusion pathways that facilitate viral cell entry [17]. These findings are consistent with the decreased infection rates consistently seen in TMPRSS2 knock-out mice and in human cohorts associated with varied genotypes or reduced expression of TMPRSS2 [18,19,20].

Although camostat has shown mixed results in COVID-19 clinical trials [10,11], it is worth noting that these studies typically screen only the parent drug; camostat is synthetic, but its structure has never been tailored to antiviral therapy. While Fujimoto et al. conducted an initial SAR investigation on nafamostat focusing on analogues featuring switched aromatic groups [14], derivatives of camostat have, to our knowledge, received only limited attention [21]. We herein begin to design, synthesise and assess such new ‘camostats’, probing additional factors including chain lengths, flexibility and hydrophobicity, seeking to demonstrate potential for structural modifications to improve antiviral potency.

Computational methods have long been instrumental in identifying drug repurposing opportunities for viral diseases [22,23]. In the TMPRSS2 binding of camostat and nafamostat, both drug-bound protease crystal structures and in silico molecular docking studies reveal two interactions that are key to complex formation [24,25]. Firstly, the *N*-terminal cationic guanidinium ‘head group’ forms a salt bridge with anionic Asp435. Once docked via this and other non-covalent attractions, the catalytic Ser441 becomes acylated by the central aromatic ester of camostat, such that the drug acts irreversibly as a suicide inhibitor [26]. However, since acylation is not instant [13,27], it seems the initial non-covalent docking is key to pre-organising the orientation and conformation of the drug within the catalytic site. Though initially driven by the electrostatic salt bridge formation, structural specificity and binding contributions from the rest of the molecule remain less clear. This uncertainty is highlighted by the considerable C-terminal ‘amide tail’ functional differences among the otherwise related guanidinium-based protease inhibitors camostat (**1a**, aliphatic dimethyl amide), nafamostat (**2**, aromatic amidinium cation) and gabexate (aromatic ethyl ester). Even the dominant natural metabolite of camostat oral administration, GBPA (4-(guanidinobenzoyloxy)phenylacetic acid, also called FOY-251), retains some of the parent drug’s inhibitory potency, despite its structure being significantly truncated at the C-terminus [3,27,28]. These structural divergences suggest that the C-terminal region may influence the binding affinity and consequent propensity for covalent adduct formation with TMPRSS2. Therefore, in the present study, modification at the C-terminus was prioritised to probe how this region contributes to protease disruption—an aspect underexplored in previous SAR initiatives.

### 1.1. Strategy of Functionality Variance

**Scheme 1:**
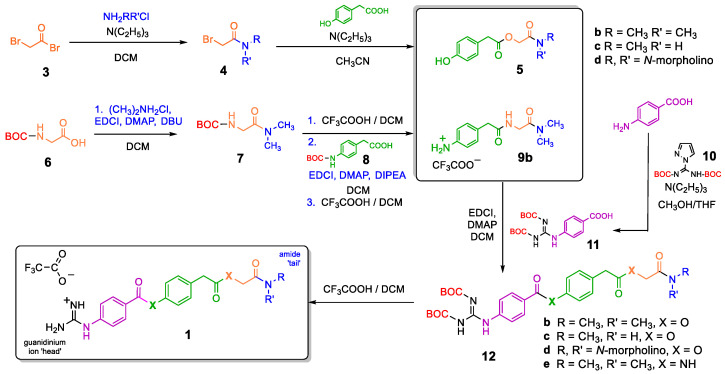
Synthesis routes to camostat trifluoroacetate (**1b**) and the three analogues (**1c**–**1e**) predicted to bind TMPRSS2 with an affinity equivalent to, or stronger than, that of camostat itself.

The apparent tolerance of structural variation at the C-terminus of TMPRSS2 inhibitors was also seen amongst several of our novel analogues during our in silico docking analysis. We therefore prioritised our initial synthetic targets to incorporate a diversity of functional group sizes and hydrophobicity, synthesising des-methyl (**1c**) and morpholino (**1d**) analogues of camostat (Scheme 1). Bis-amide **1e**, effectively a tripeptide, was devised in order to shed light on the role of irreversible acylation in camostat’s mode of action; the amide replacement is considerably more stable than the parent drug’s ester unit towards the nucleophilic serine of the protease binding pocket.

**Figure 2:**
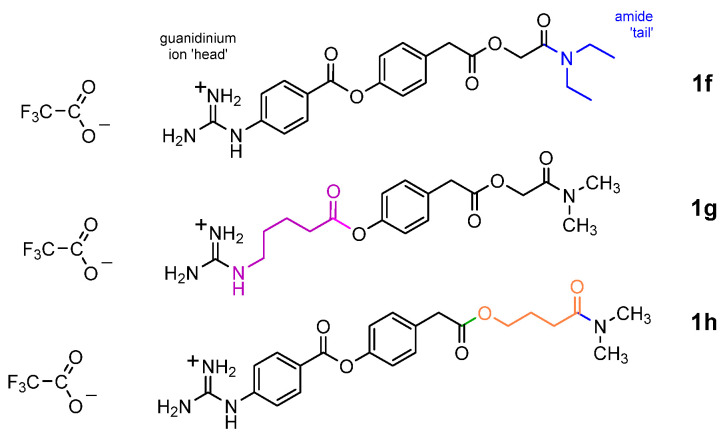
The molecular structures of additional camostat analogues included in the initial docking studies, which feature flexible or extended building blocks. The structure of each analogue differs from that of camostat (**1b**) by the colour-highlighted residue only (blue = diethylamide ‘tail’, purple = 5-aminopentanoyl residue, orange = 4-hydroxybuanoyl residue).

Three other camostat analogues (**1f** to **1h**) were additionally devised and included in our initial in silico screening to probe the effects of incorporating greater conformational flexibility at different points along the backbone of the drug structure (Figure 2). Analogue **1f** featured an alternative C-terminal *N*-diethyl amide modification, which increases the hydrophobicity and conformational flexibility of camostat’s C-terminal dimethyl amide. Analogue **1g** replaced the *N*-terminal aromatic residue with a more flexible and saturated 5-amino-pentanoyl unit of deliberately matching chain length. Finally, **1h** was conceived to elongate the molecule by replacing its glycolyl unit with a 4-hydroxy-butanoyl residue. These modifications were selected to explore whether increased backbone flexibility or the introduction of polar functional groups could enhance binding adaptability within the TMPRSS2 active site. In particular, **1g** and **1h** were designed to test the effects of increased flexibility and whether an extended linker (in **1h**) may improve molecular accommodation (binding pocket ‘snugness-of-fit’), thereby altering inhibitory potency or pharmacokinetic properties, such as solubility or metabolic stability.

### 1.2. In Vitro Bioassay

To complement our docking studies, we measured TMPRSS2 inhibition of the synthesised camostat analogues via in vitro assays featuring a doubly tagged substrate featuring a fluorophore–quencher pair, an oligopeptide mimic of the SARS-CoV-2 spike protein S2′ site (PSKPSKR↓SFIEDL). Unlike other parts of the spike protein, the sequence around S2′, at which cleavage selectively occurs to the C-side of Arg 815 as part of the viral membrane fusion pathway, was conserved across all the most virulent SARS-CoV-2 variants that emerged during the COVID-19 pandemic [29,30].

### 1.3. Organic Synthesis Considerations

Camostat molecules can be considered, retrosynthetically, to comprise four structural units, a modular construct that lends itself to the incorporation of structural variety through connected ester or amide bonds. Our five-step synthetic route to camostat analogues draws on various approaches in the literature and is further tailored to allow purifications by partition extraction and precipitation (Scheme 1). The synthesis of the *N*-terminal guanidinylated building block **11** takes advantage of carboxamidine **10**, which can convert a variety of amines into their di-BOC-protected *N*-guanidino analogues under relatively mild conditions by modifying the procedure outlined by Riches et al. [31]. Though we have observed a wide synthetic scope for this *N*-guanidinylation, our initial pilot study opted to initially retain 4-aminobenzoic acid at the *N*-terminus. Since it is common to both the parent drug camostat and nafamostat, it is likely that the rigidity of this aromatic residue fosters stabilisation of the guanidinium’s salt bridge interaction within the protease binding pocket. Moreover, maintaining this structural consistency allowed clearer comparison of C-terminal variation effects.

For the C-terminal derivatives, the synthesis of each derivative’s 2-bromocarboxamide (**4**) building block involved an initial amidation of bromoacetyl bromide (**3**) through minor modification of the literature precedents [32,33]. For more volatile amines, this nucleophilic acyl substitution necessitated the corresponding hydrochloride salts (affording **4b** and **4c**), whereas the corresponding free secondary amine sufficed to generate the morpholino derivative **4d**. The yields were generally low (<50%), but amide products were obtained cleanly with only simple aqueous washing. Our initial amidation attempts involving acid chloride activations of 2-bromoacetic acid and 2-bromoacetyl chloride, by contrast, gave an undesirable mixture of products. The substitution of 4-hydroxyphenylacetic acid was then completed to incorporate the central building block of camostat, affording esterified phenols **5b**–**d** by modifying the approach taken by Ono Pharmaceuticals [34]. Among the common polar aprotic solvents that best favour such S_N_2 displacements, we also selected acetonitrile since it allowed hot reflux heating, to ensure a rapid reaction time, but enough volatility for subsequent convenient removal in vacuo prior to work up. Carbodiimide-mediated coupling between **11** and **5b**–**d** or the protected precursor of **9** to install the aromatic ester and amide linkages afforded di-BOC-protected camostats (**12b**–**e**) in high yields. Final deprotection in anhydrous trifluoroacetic acid (TFA)-solution afforded the guanidinium trifluoroacetate salts (**1b**–**e**), with precipitation from ether maintaining product purity. Although camostat is delivered as a mesylate (methanesulfonate) salt for therapeutic purposes, the identity of the counteranion in protease assays is expected to be relatively insignificant; it is only the cation that binds the protease catalytic site, and, as a consequence, this initial study proceeded without exchanging the TFA anion. Indeed, some proven TMPRSS2 inhibitors, e.g., the peptidomimetic MI-432 [35], are made commercially available as the TFA salt [36]. For the synthesis of bis-amide camostat **1e**, the chain was extended using standard BOC-protected amino acid and classical solution-phase peptide synthesis. BOC-protected aromatic amino acid **8** was generated efficiently via a procedure patented by Ipsen [37]. C-terminal amidations were completed with conventional carbodiimide coupling to give intermediates **7** and **9a**, with final coupling and BOC-deprotection affording bis-amide guanidinium salt **1e**.

## 2. Results and Discussion

### 2.1. In Silico Docking

Our Autodock Vina data predict molecular interactions for camostat, displaying interactions analogous to those published previously [25], with a computed (top pose, i.e., strongest binding conformation) binding affinity of −6.6 kcal/mol (and −6.4 ± 0.2 kcal/mol for the average of the top three poses). In our dockings, two of our proposed C-terminal amide-modified derivatives (**1c** and **1d**) bind TMPRSS2 non-covalently with affinities slightly greater (−7.0 kcal/mol) than that of camostat itself (Table 1).

The *N*-diethyl amide tail of **1f**, by contrast, is predicted to slightly reduce protease binding. The hydrophobicity of the *N*-diethyl amide lies between that of the *N*-dimethyl and *N*-morpholino amides, so its comparatively weaker binding (−6.0 kcal/mol) may be attributable to additional disruptive conformational flexibility incurred by two separate untethered *N*-alkyl groups. Collectively, these findings point to a considerable scope for further structural fine-tuning of the drug’s amide ‘tail’ region in future compound libraries.

Bis-amide **1e** was predicted to non-covalently bind TMPRSS2 with similar affinity as camostat itself, suggesting that the swap-in of hydrogen bonding NH groups does not disrupt complex formation. It is likely that the increased resonance-induced planarity of the peptide linker replacements contributes to conformational rigidity, compared to the more flexible ester derivatives, perhaps helping the bis-amide ligand settle tighter in the binding pocket.

The noticeably weaker complex formed by **1g** illustrates well the importance of rigidity in the guanidinium hosting unit [38]. The *N*-terminal linker length of **1g** was designed to match that of its equivalent in camostat. The far higher rotatable bond count and consequent entropic freedom of the alkyl chain clearly disrupt complex formation, implying a role for rigidity in the aromatic unit’s contribution to the tighter binding camostat. A similar but smaller drop in binding energy is seen when camostat’s C_2_ glycolic acid is swapped for a longer, more flexible C_4_ equivalent in **1h**, but, in this case, at last two possible factors, i.e., the linker’s greater flexibility and increased overall length, need to be separated out in determining requirements for optimum binding, a potential focus of future SAR work.

**Figure 3:**
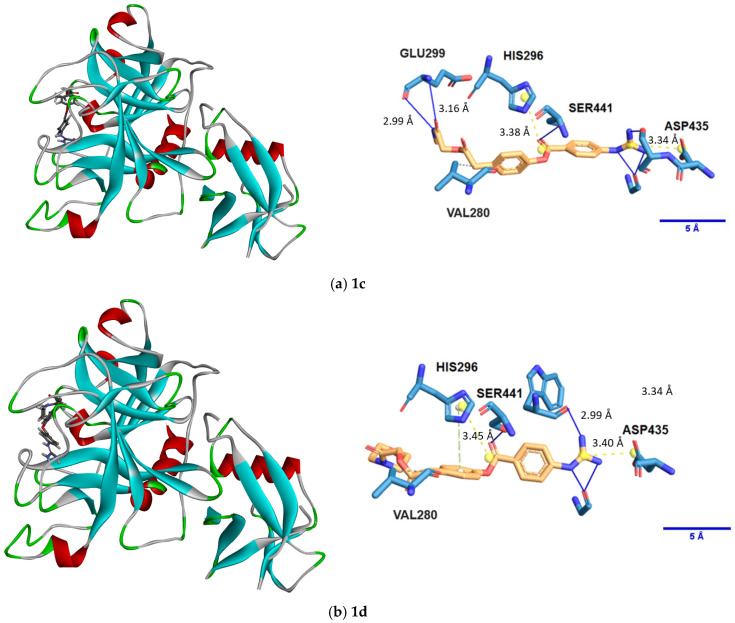
Complexes formed between TMPRSS2 and two strongly binding camostat analogues, (**a**) **1c** (above) and (**b**) **1d** (below), as determined by molecular docking. Left: Pose views for the top-ranked poses of each enzyme–ligand non-covalent complex. Right: Key non-covalent interactions within the TMPRSS2 catalytic binding pocket, shown for each enzyme–ligand complex. Drug analogue carbon skeletons are shown in gold, with relevant sections of protease residues in blue. In both complexes, the guanidinium ion salt bridge to Asp435 (far right) is indicated, which forms prior to acylation of Ser441 by the aromatic ester.

Modelling revealed that both of the strongest binding analogues, **1c** and **1d**, form similar interactions with the protease binding pocket residues (Figure 3). Consistently evident are the salt bridge to Asp435 and consequent preorganisation of the electrophilic aromatic ester adjacent to Ser441. Further along the drug scaffold, **1c** also forms hydrogen bonds from its secondary amide ‘tail’ to Glu299 and a nearby hydrophobic contact with Val280. By contrast, **1d** appears to lack hydrogen bonding to Glu299, probably on account of the absence of an NH in the morpholino (tertiary) amide. Although this absence is interpreted as a loss of a stabilising interaction, the overall docking score of **1d** remains comparable to that of **1c**. This suggests that the morpholino ring may confer compensatory advantages, such as an entropy-driven enhancement of the hydrophobic contact area.

### 2.2. Synthesis

We prioritised the synthesis of the docking-predicted stronger binding camostat analogues. The structures and purities of the final guanidinium TFA salt products were confirmed by LC-MS and ^1^H-NMR, with DMSO-d_6_ proving to be (with a few exceptions) the most effective solvent for tracking and reliable integration of NH signals. In particular, the two guanidinium NH signals proved to be of remarkably consistent and compound-specific chemical shift for each of the di-BOC precursor intermediates. The final salts (**1b**–**1e**) all feature a distinctive 4H broad singlet around δ7.7, characteristic of the resonance-stabilised guanidinium cation.

Our ester and amide couplings were performed with 1-ethyl-3-(3-dimethylaminopropyl)carbodiimide (EDCI) and 4-dimethylaminopyridine (DMAP) included *N*,*N*-diisopropylethylamine (DIPEA), but we later found that the tertiary amine can be omitted if DMAP is used in slight excess, thereby serving dual purpose roles as both base and acylation accelerant catalyst.

Phenols (**5b**–**d**) could typically be precipitated out by aqueous dilution of the organic residue and collected; thus, whenever a phenol intermediate product remained dissolved at this stage, it was instead successfully extracted into ethyl acetate.

The work up following the synthesis of esters **12b**–**d** was found to necessitate a strong (20% *w*/*v*) sodium carbonate solution; weaker bicarbonate (p*K*a ≈ 10) is insufficiently basic to completely ionise the residual phenol intermediate (**5b**–**d**, p*K*a ≈ 9–11) starting material for extractive wash out. By contrast, even dilute sodium hydroxide, though sufficiently alkaline to ensure phenol deprotonation, can hydrolytically cleave the product’s sensitive aliphatic ester group, leading to a lack of product isolation.

### 2.3. Protease Inhibition Assays

**Figure 4:**
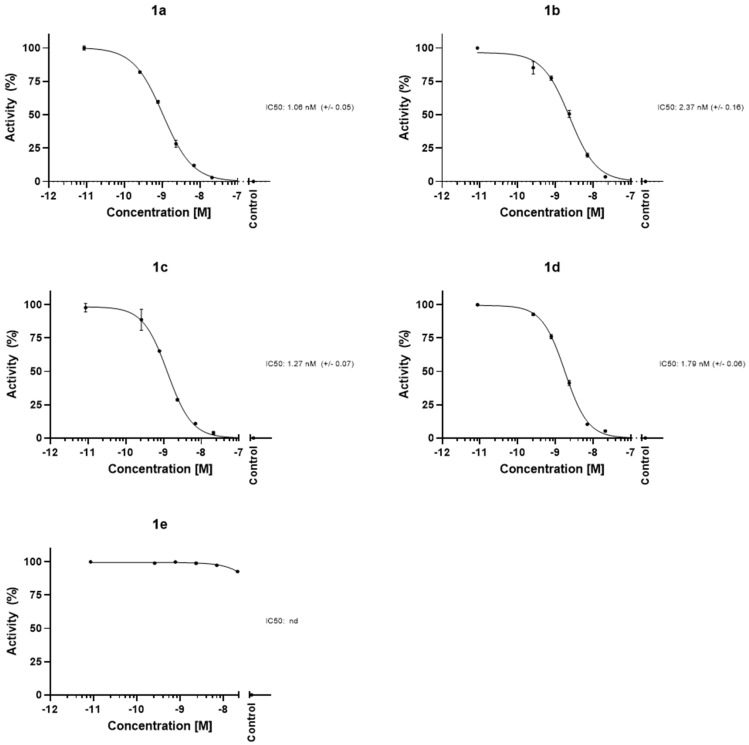
In vitro TMPRSS2 enzyme inhibition activity to determine IC_50_ values (summarised in Table 2) for five camostat derivatives (**1a** to **1e**) in the presence of TMPRSS2 protease. All derivatives display nanomolar binding affinities except bis-amide analogue **1e**.

The fluorescence assay data confirmed that camostat binds TMPRSS2 with nanomolar affinity, in agreement with other studies [13,27,28] (Figure 4). Our data found minimal difference between the binding strength of a reference sample of camostat mesylate salt (**1a**) and our synthesised camostat TFA salt (**1b**), a finding consistent with the counterion remaining uninvolved in complexation and separated from the enzyme binding pocket.

Any TMPRSS2 inhibition occurring for bis-amide **1e** fell out of the range of the assay and was deemed to be significantly less potent. This apparent lack of complex formation is consistent with the need for Ser441 acylation in camostat’s mode of action, as similarly observed in cell-based screens of a nafamostat amide derivative [14].

## 3. Materials and Methods

### 3.1. Molecular Docking

Molecular docking was performed using the unliganded model structure of TMPRSS2 protease with Protein Data Bank (PDB) ID: 7MEQ [13,25] (accessed 12 May 2025), which was set as the docking target. Camostat and the synthetic analogues herein described were used as ligands.

Ligand structures were drawn using the molecular editor of the 1-Click Docking tool available on the Mcule.com platform (Mcule Inc.—1-Click Mcule, Palo Alto, CA, USA. Available online: https://mcule.com (accessed on 15 June 2025)) [39,40,41]. This online platform features an embedded version of AutoDock Vina [42] to carry out molecular docking operations. The two-dimensional structure of each compound was drawn directly in the molecular editor provided within the 1-Click Docking tool. Mcule.com uses AutoDock Tools to prepare both the ligand and the target protein for the docking process. Docking runs were performed using the unliganded model structure of TMPRSS2 protease with the atomic coordinates of the binding site being set as X: −8.55, Y: −2.05 and Z: 16.07, based on previously reported literature values for the binding centre [25], and the binding site size being set to 22 Å. The ligand–enzyme complexes with the lowest binding energy poses were then evaluated and visualised using Discovery Studio Visualizer v21.1.0.20298. Protein–ligand interaction diagrams were generated using PLIP (Protein–Ligand Interaction Profiler; available online: https://plip-tool.biotec.tu-dresden.de/plip-web/plip/index (accessed on 15 June 2025)), as reported in other examples in the literature [43]. The scores obtained in our molecular dockings represent the predicted affinity of the docked ligands to TMPRSS2.

### 3.2. Organic Synthesis Procedures

*N*,*N*′-Di-Boc-1*H*-pyrazole-1-carboxamidine (**10**) and bromoacetyl bromide, 4-aminobenzoic acid, 4-aminophenylacetic acid, 4-hydroxyphenylacetic acid, glycine, triethylamine, EDCI, DMAP, DIPEA, di-*tert*-butyl dicarbonate and trifluoroacetic were purchased from Fluorochem Ltd. (Glossop, UK). Dimethylammonium hydrochloride and methylammonium hydrochloride were purchased from ThermoFisher Scientific Inc. UK (Altrincham, UK) and morpholine from Alfa Aesar UK (Loughborough, UK). The progress and success of chemical reactions were monitored by ^1^H-NMR and reversed-phase LC-MS (Figures S1–S18). NMR spectra were obtained using a JEOL ECZR 400 (^1^H 399.78 MHz) or JEOL ECA 500 (^1^H 500.16 MHz) spectrometer (JEOL Ltd., Tokyo, Japan) and are reported relative to the residual solvent reference chemical shifts. LC-MS analysis was performed on a Shimadzu LC-2020 system featuring a Shimadzu LC-2050C chromatograph (Shimadzu UK Limited, Milton Keynes, UK) in conjunction with a reversed-phase column (H_2_O-MeCN; a 2-min MeCN hold extended run time for all di-BOC compounds to ensure elution).

Synthesis of **4b**: A slurry of dimethylammonium hydrochloride (4.86 g, 60 mmol) and triethylamine (9.2 mL, 70 mmol) in dry dichloromethane (30 mL) was cooled to 0 °C. Bromoacetyl bromide (3.50 mL, 40 mmol) in dry dichloromethane (15 mL) was added dropwise from an air-sealed pressure-equalising dropping funnel over 1 h with stirring. The mixture was then stirred for a further 1 h at 0 °C and 3 h at 25 °C. The mixture was then washed (water → brine), dried (MgSO_4_) and evaporated in vacuo to leave the product as a brown mobile liquid (2.63 g, 40%) that was used without further purification.

^1^H NMR (500 MHz, DMSO-d_6_) δ 4.35 and δ 4.10 (rotamer singlet pair, 2H), 3.02 and 2.99 (rotamer singlet pair, 3H), 2.84 (rotamer singlet pair, 3H).

^1^H NMR (500 MHz, CDCl_3_) δ 4.09 and δ 3.87 (rotamer singlet pair, 2H), 3.11 (s, 3H, CH_3_), 3.00 (s, 3H, CH_3_). LC-MS: *m*/*z* 165.80, 167.85 [M + H]^+^ isotope (Br) pair.

Synthesis of **4c**: A slurry of methylammonium hydrochloride (5.40 g, 80 mmol) and anhydrous potassium carbonate (22 g, 160 mmol) in dry dichloromethane (125 mL) was cooled to 0 °C. Bromoacetyl bromide (7.00 mL, 80 mmol) in dry dichloromethane (25 mL) was added dropwise from an air-sealed pressure-equalising dropping funnel over 1 h with stirring. The mixture was then stirred for a further 1 h at 0 °C and 3 h at 25 °C. The mixture was then washed (water → brine), dried (MgSO_4_) and evaporated in vacuo to leave the product as a white solid (5.61 g, 46%) that was used without further purification.

^1^H NMR (500 MHz, DMSO-d_6_) δ 8.20 (br s, 1H, NH), 3.83 (s, 2H, CH_2_), 2.61 (d, *J* = 4.7 Hz, 3H, CH_3_). LC-MS: *m*/*z* 151.8, 153.8 [M + H]^+^ isotope (Br) pair.

Synthesis of **4d**: A solution of morpholine (1.42 mL, 16.5 mmol) in dry dichloromethane (15 mL) was cooled to −10 °C by a rock salt–ice bath. Bromoacetyl bromide (870 μL, 10 mmol) in dry dichloromethane (5 mL) was added dropwise from an air-sealed pressure-equalising dropping funnel over 1 h with stirring. The mixture was then stirred for a further 1 h at 0 °C and 4 h at 25 °C. The mixture was then washed (0.5 M citric acid → brine), dried (MgSO_4_) and evaporated in vacuo to leave the product, a runny pale pink oil (1.12 g, 54%), that was used without further purification.

^1^H NMR (500 MHz, CDCl_3_) δ 3.75 and 3.70 (rotamer triplet pair, *J* = 4.8 Hz, 4H, CH_2_), 3.64 and 3.53 (rotamer triplet pair, *J* = 4.8 Hz, 4H, CH_2_). LC-MS: *m*/*z* 207.9, 209.9 [M + H]^+^ isotope (Br) pair.

Synthesis of **5b**: *N*,*N*-dimethyl-2-bromoacetamide (**4b**, 685 mg, 4.1 mmol) and 4-hydroxyphenylacetic acid (593 mg, 3.9 mmol) were dissolved in acetonitrile (12 mL). Triethylamine (740 μL 5.3 mmol) was then added, and the mixture was refluxed under a findenser (6 h). Following the removal of most of the solvents in vacuo, cold water (10 mL) was added to the residue and refrigerated (2 h). The precipitated product was collected by filtration and oven dried (50–55 °C, 4 h) to afford the ester-coupled product as a pale brown solid (484 mg, 52%).

^1^H NMR (500 MHz, DMSO-d_6_) δ: 9.29 (s, 1H, OH), 7.07 (d, 2H, *J* = 8.5 Hz, ArH), 6.69 (s, 2H, *J* = 8.5 Hz, ArH), 4.75 (s, 2H, CH_2_), 3.60 (s, 2H, CH_2_), 2.89 (s, 3H, CH_3_), 2.81 (s, 3H, CH_3_). LC-MS: *m*/*z* 238.10 [M + H]^+^, 236.10 [M − H]^−^.

Synthesis of **5c**: *N*-methyl-2-bromoacetamide (**4c**, 385 mg, 2.53 mmol) and 4-hydroxyphenylacetic acid (502 mg, 3.30 mmol) were dissolved in DMF (2.5 mL). DIPEA (620 μL 3.55 mmol) was then added, and the mixture was heated under a findenser (60 °C, 16 h). The mixture was then diluted with ethyl acetate, washed (0.2 M HCl → sat. NaHCO_3_ → brine) and then dried (MgSO_4_) and evaporated in vacuo to afford the ester-coupled product as an off-white solid (205 mg, 36%).

^1^H NMR (500 MHz, DMSO-d_6_) δ: 9.30 (s, 1H, OH), 7.91 (br s, 1H, NH), 7.07 (d, 2H, *J* = 8.5 Hz, ArH), 6.70 (s, 2H, *J* = 8.5 Hz, ArH), 4.43 (s, 2H, OCH_2_), 3.63 (s, 2H, ArCH_2_), 2.61 (d, *J* = 6.5 Hz, 3H, CH_3_). LC-MS: *m*/*z* 224.1 [M + H]^+^, 246.0 [M + Na]^+^, 222.0 [M − H]^−^, 445.1 [2M − H]^−^.

Synthesis of **5d**: *N*-morpholino-2-bromoacetamide (**4d**, 650 mg, 3.125 mmol) and 4-hydroxyphenylacetic acid (464 mg, 3.05 mmol) were dissolved in acetonitrile (10 mL). Triethylamine (740 μL 5.3 mmol) was then added, and the mixture was refluxed under a findenser (6 h). Following the removal of most of the solvents in vacuo, cold water (10 mL) was added to the residue and refrigerated (2 h). The precipitated product was collected by filtration and oven dried (50–55 °C, 4 h) to afford the ester-coupled product as a pale brown solid (522 mg, 61%).

^1^H NMR (500 MHz, DMSO-d_6_) δ: 9.29 (s, 1H, OH), 7.91 (br s, 1H, NH), 7.07 (d, 2H, *J* = 8.5 Hz, ArH), 6.69 (s, 2H, *J* = 8.5 Hz, ArH), 4.78 (s, 2H, OCH_2_), 3.60 (s, 2H, ArCH_2_), 3.54 (m, 4H, CH_2_), 3.40 (2 × m, 4H, CH_2_). LC-MS: *m*/*z* 279.95 [M + H]^+^, 301.85 [M + Na]^+^, 581.25 [2M + Na]^+^.

Synthesis of **7**: BOC-Glycine (**6**, 7.0 g, 40 mmol) was taken up in DCM (40 mL) and slurried with dimethylammonium hydrochloride (4.88 g, 60 mmol). EDCI (9.2 g, 48 mmol) and DMAP (2.44 g, 20 mmol) were then added, followed by DBU (10.4 mL, 70 mmol), and the mixture was stirred for 24 h. The mixture was then diluted four-fold with ethyl acetate, washed (0.2 M HCl → 10% Na_2_CO_3_ → brine) and then dried (MgSO_4_) and evaporated in vacuo to leave the tertiary amide product **7** as a white solid (2.46 g, 30%).

^1^H NMR (400 MHz, DMSO-d_6_) δ: 6.64 (t, 1H, NH), 3.74 (d, 2H, *J* = 6.0 Hz CH_2_), 2.92 (s, 3H), 2.81 (s, 3H), 1.37 (s, 9H, ^t^Bu). LC-MS: *m*/*z* 203.0 [M + H]^+^, 147.0 [M − ^t^Bu + H]^+^, 427.15 [2M + Na]^+^.

Synthesis of **8**: (BOC-4-aminophenylacetic acid): To a solution of 4-aminophenylacetic acid (906 mg, 6.0 mmol) and anhydrous sodium carbonate powder (2.25 g, 21 mmol) in water (10 mL) was slowly added di-*tert*-butyl dicarbonate (BOC_2_O, 1.83 g, 8.4 mmol) in THF (20 mL) at 0 °C with rapid stirring. The mixture was gradually allowed to warm to room temperature and stirred vigorously overnight. The THF was then removed in vacuo, and the remaining aqueous solution was diluted with water (15 mL), acidified to pH 2 via dropwise addition of HCl (conc). The resulting thin suspension was thrice extracted with ethyl acetate, and the combined organic layers were dried (MgSO_4_) and evaporated in vacuo to afford the product as a white solid (1.29 g, 85%).

^1^H NMR (500 MHz, DMSO-d_6_) δ: 9.27 (s, 1H, NH), 7.37 (d, 2H, *J* = 8.5 Hz, ArH), 7.12 (d, 2H, *J* = 8.5 Hz, ArH), 3.46 (s, 2H, CH_2_), 1.47 (s, 9H, ^t^Bu). LC-MS: *m*/*z* 269.0 [M + NH_4_]^+^, 274.0 [M + Na]^+^, 501.1 [2M − H]^−^.

Synthesis of **9**: BOC-protected amine **7** (1.375 mmol) was stirred in TFA/DCM (2:3 *v*/*v*, 3 mL) for 2.5 h (until LC-MS confirmed the completion of BOC cleavage). Following extensive removal of all volatiles in vacuo, the mixture was then taken up in DCM (5 mL). BOC-4-aminophenylacetic acid (**8**, 345 mg, 1.375 mmol) was then added, followed by EDCI (368 mg, 1.92 mmol), DMAP (335 mg, 2.75 mmol) and DIPEA (244 μL, 1.40 mmol), and the mixture was stirred for 20 h. The mixture was then diluted four-fold with ethyl acetate, washed (0.1 M HCl → sat. NaHCO_3_ → brine) and then dried (MgSO_4_) and evaporated in vacuo to afford **9a** (the BOC-protected precursor of ammonium salt **9b**) as a fluffy white powder (278 mg, 60%).

^1^H NMR (500 MHz, DMSO-d_6_) δ: 9.27 (br s, 1H, NHAr), 7.98 (t, *J* = 5.4 Hz, 1H, NH), 7.35 (d, 2H, *J* = 8.5 Hz, ArH), 7.15 (d, 2H, *J* = 8.5 Hz, ArH), 3.90 (d, *J* = 5.4 Hz, 2H, NCH_2_), 3.41 (s, 2H, ArCH_2_), 2.92 (s, 3H, CH_3_), 2.82 (s, 3H, CH_3_), 1.46 (s, 9H, ^t^Bu). LC-MS: *m*/*z* 336.25 [M + H]^+^, 358.20 [M + Na]^+^.

BOC-protected amide **9a** was stirred in TFA/DCM (1:1 *v*/*v*, 3 mL) for 4 h, before solvents were evaporated. Further azeotropic co-evaporation with toluene yielded trifluoroacetate salt **9b**.

Synthesis of **11**: 4-Aminobenzoic acid (6.66 g, 48.6 mmol) and *N*,*N*′-di-BOC-1H-pyrazole-1-carboxamidine (**10**, 7.55 g, 24.3 mmol) were taken up in methanol (80 mL). Triethylamine (10 mL, 73 mmol) was then slowly added, and the mixture was heated under a findenser (52 °C, 18 h). The solvents were then removed in vacuo, and the residue was redissolved in dichloromethane. Following aqueous washes (0.2 M HCl → brine), the organic layer was dried (MgSO_4_) and evaporated in vacuo to yield the product as a white solid (8.10 g, 87.5%) that was used without further purification.

^1^H NMR (500 MHz, DMSO-d_6_) δ: 10.18 (br s, 1H, NH), 7.90 (d, 2H, *J* = 6.8 Hz, ArH), 7.67 (br s, 2H, ArH), 1.45 (s, 18H, ^t^Bu). LC-MS: *m*/*z* 380.1 [M + H]^+^.

Synthesis of **12b**: Di-BOC-guanidinobenzoic acid (**11**, 392 mg, 1.033 mmol) and phenol **5b** (213 mg, 0.899 mmol) were taken up in DCM (3 mL). EDCI (288 mg, 1.50 mmol) and DMAP (305 mg, 2.50 mmol) were then added, followed by DIPEA (350 μL, 2.00 mmol), and the mixture was stirred for 24 h. The mixture was then diluted four-fold with ethyl acetate, washed (0.2 M citric acid → 10% Na_2_CO_3_ → brine) and then dried (MgSO_4_) and evaporated in vacuo to give the product as an off-white foamy solid (430 mg, 80%). n.b., for the alkali wash, NaHCO_3_ was found to be insufficiently basic to remove the phenol.

^1^H NMR (500 MHz, DMSO-d_6_) δ: 11.21 (s, 1H, NH), 10.24 (s, 1H, NH), 8.10 (d, 2H, *J* = 8.8 Hz, ArH), 7.81 (d, 2H, *J* = 8.8 Hz, ArH), 7.39 (d, 2H, *J* = 8.7 Hz, ArH), 7.23 (d, 2H, *J* = 8.7 Hz, ArH), 4.81 (s, 2H, OCH_2_), 3.81 (s, 2H, ArCH_2_), 2.91 (s, 3H, CH_3_), 2.82 (s, 3H, CH_3_), 1.51 (s, 9H, ^t^B), 1.43 (s, 9H, ^t^Bu). LC-MS: *m*/*z* 599.30 [M + H]^+^, 499.20 [M − BOC + H]^+^, 399.10 [M − 2BOC + H]^+^, 597.35 [M − H]^−^.

Synthesis of **12c**: Di-BOC-guanidinobenzoic acid (**11**, 246 mg, 0.65 mmol) and phenol **5c** (125 mg, 0.56 mmol) were taken up in DCM (3 mL). EDCI (172 mg, 0.90 mmol) and DMAP (182 mg, 1.50 mmol) were then added, followed by DIPEA (406 μL, 2.33 mmol), and the mixture was stirred for 24 h. The mixture was then diluted four-fold with ethyl acetate, washed (0.2 M citric acid → 10% Na_2_CO_3_ → brine) and then dried (MgSO_4_) and evaporated in vacuo to give the product as a white solid (317 mg, 97%).

^1^H NMR (500 MHz, DMSO-d_6_) δ: 11.21 (s, 1H, NH), 10.25 (s, 1H, NH), 8.10 (d, 2H, *J* = 8.8 Hz, ArH), 7.95 (br m, 1H, NHMe), 7.82 (d, 2H, *J* = 8.8 Hz, ArH), 7.38 (d, 2H, *J* = 6.5 Hz, ArH), 7.23 (d, 2H, *J* = 8.7 Hz, ArH), 4.49 (s, 2H, OCH_2_), 3.83 (s, 2H, ArCH_2_), 2.63 (d, *J* = 4.6 Hz, 3H, CH_3_), 1.51 (s, 9H, ^t^Bu), 1.43 (s, 9H, ^t^Bu). LC-MS: *m*/*z* 585.25 [M + H]^+^, 607.15 [M + Na]^+^, 583.20 [M − H]^−^.

Synthesis of **12d**: Di-BOC-guanidinobenzoic acid (**11**, 531 mg, 1.40 mmol) and phenol **5d** (335 mg, 1.20 mmol) were taken up in DCM (4 mL). EDCI (384 mg, 2.00 mmol) and DMAP (390 mg, 3.20 mmol) were then added, followed by DIPEA (870 μL, 5.00 mmol), and the mixture was stirred for 24 h. The mixture was then diluted four-fold with ethyl acetate, washed (0.2 M citric acid → 10% Na_2_CO_3_ → brine) and then dried (MgSO_4_) and evaporated in vacuo to give the product as a foamy solid (724 mg, 94%).

^1^H NMR (500 MHz, DMSO-d_6_) δ: 11.22 (s, 1H, NH), 10.24 (s, 1H, NH), 8.10 (d, 2H, *J* = 8.8 Hz, ArH), 7.82 (br d, 2H, *J* = 8.8 Hz, ArH), 7.39 (d, 2H, *J* = 6.5 Hz, ArH), 7.23 (d, 2H, *J* = 8.7 Hz, ArH), 4.84 (s, 2H, CH_2_), 3.82 (s, 4H, CH_2_), 3.42 and 3.37 (pair of rotamer quartets, 8H, CH_2_), 1.47 (br s, 18H, ^t^Bu). LC-MS: *m*/*z* 641.35 [M + H]^+^, 541.20 [M − BOC + H]^+^, 441.10 [M − 2BOC + H]^+^, 639.10 [M − H]^−^.

Synthesis of **12e**: Trifluoroacetate salt **9b** (1.047 mmol) was taken up in DCM (6 mL), and di-BOC-guanidinobenzoic acid (**11**, 397 mg, 1.047 mmol) was added. Following dissolution, EDCI (307 mg, 1.60 mmol), DMAP (342 mg, 2.80 mmol) and DIPEA (700 μL, 4.00 mmol) were added, and the solution was stirred for 24 h. The mixture was then diluted four-fold with ethyl acetate, washed (0.5 M citric acid → sat. NaHCO_3_ → brine) and then dried (MgSO_4_) and evaporated in vacuo to give the amide product (476 mg, 76%).

^1^H NMR (500 MHz, DMSO-d_6_) δ: 11.33 (s, 1H, NH), 10.17 (s, 1H, NH), 10.16 (s, 1H, NH), 8.05 (t, *J* = 5.4 Hz, 1H, NH), 7.94 (d, *J* = 8.8 Hz, 2H, ArH), 7.73 (d, *J* = 8.8 Hz, 2H, ArH), 7.68 (d, *J* = 8.6 Hz, 2H, ArH), 7.25 (d, *J* = 8.6 Hz, 2H, ArH), 3.92 (d, *J* = 5.4 Hz, 2H, NCH_2_), 3.47 (s, 2H, ArCH_2_), 2.93 (s, 3H, CH_3_), 2.83 (s, 3H, CH_3_), 1.52 (s, 9H, ^t^Bu), 1.42 (s, 9H, ^t^Bu). LC-MS: *m*/*z* 597.45 [M + H]^+^, 595.10 [M − H]^−^.

Synthesis of **1b**–**1e**: Each di-BOC-protected camostat analogue (**12**) was stirred in TFA/DCM (1:3 *v*/*v*, 1–3 mL) for 3–4 h (or until LC-MS confirmed the complete cleavage of both BOC groups). The volatiles were then immediately and thoroughly evaporated in vacuo at room temperature before ice-cold ether (10 mL) was slowly added to the residue with swirling. Following further refrigeration (2–3 h), the typically off-white precipitate was triturated with the mother liquor from which it formed, swirled and then allowed to settle (1 h). The resulting pellet of guanidinium trifluoroacetate salt was then isolated by gradual decanting of the solventfollowed by drying under high vacuum (2 h, or until NMR confirmed the complete ether removal). The samples were kept at −20 °C for long-term storage.

Synthesis of **1b**: (**12b**, 200 mg, 0.3344 mmol) was deprotected as described above to afford salt **1b** as a white solid (170 mg, 99%).

^1^H NMR (500 MHz, DMSO-d_6_) δ: 10.35 (s, 1H, NHAr), 8.16 (d, *J* = 8.8 Hz, 2H, ArH), 7.88 (br s, 4H, NH), 7.43 (d, *J* = 8.8 Hz, 2H, ArH), 7.40 (d, *J* = 8.6 Hz, 2H, ArH), 7.23 (d, *J* = 8.6 Hz, 2H, ArH), 4.81 (s, 2H, OCH_2_), 3.82 (s, 2H, ArCH_2_), 2.91 (s, 3H, CH_3_), 2.82 (s, 3H, CH_3_). LC-MS: *m*/*z* 399.1 [M + H]^+^, 397.10 [M − H]^−^.

Synthesis of **1c**: (**12c**, 200 mg, 0.3424 mmol) was deprotected as described above to afford salt **1c** as a white solid (170 mg, 100%).

^1^H NMR (500 MHz, DMSO-d_6_) δ: 10.23 (s, 1H, NH), 8.16 (d, *J* = 8.8 Hz, 2H, ArH), 7.99 (br m, 1H, NHMe), 7.82 (br s, 4H, NH), 7.43 (d, *J* = 8.8 Hz, 2H, ArH), 7.40 (d, *J* = 8.7 Hz, 2H, ArH), 7.23 (d, *J* = 8.7 Hz, 2H, ArH), 4.49 (s, 2H, OCH_2_), 3.84 (s, 2H, ArCH_2_), 2.62 (d, *J* = 4.6 Hz, 3H, CH_3_). LC-MS: *m*/*z* 385.0 [M + H]^+^, 392.9 [M − H]^−^.

Synthesis of **1d**: (**12d**, 90 mg, 0.1406 mmol,) was deprotected as described above to afford salt **1d** as a white solid (84 mg, 98%).

^1^H NMR (500 MHz, DMSO-d_6_) δ: 10.26 (s, 1H, NH), 8.16 (d, *J* = 8.8 Hz, 2H, ArH), 7.84 (br s, 4H, NH), 7.43 (br d, *J* = 8.8 Hz, 2H, ArH), 7.40 (d, *J* = 8.7 Hz, 2H, ArH), 7.23 (d, *J* = 8.7 Hz, 2H, ArH), 4.85 (s, 2H, OCH_2_), 3.82 (s, 4H, ArCH_2_), 3.56 and 3.40 (pair of rotamer quartets, 8H, CH_2_). LC-MS: *m*/*z* 441.0 [M + H]^+^, 439.1 [M − H]^−^.

Synthesis of **1e**: (**12e**, 25 mg, 0.0419 mmol) was deprotected as described above to afford salt **1e** as a white solid (21 mg, 96%).

^1^H NMR (500 MHz, DMSO-d_6_) δ: 10.21 (s, 1H, NHAr), 10.00 (s, 1H, NHAr), 8.04 (d, *J* = 8.6 Hz, 2H, ArH), 8.02 (t, *J* = 5.4 Hz, 1H, NH), 7.68 (d, *J* = 8.6 Hz, 2H, ArH), 7.37 (d, *J* = 8.6 Hz, 2H, ArH), 7.26 (d, *J* = 8.6 Hz, 2H, ArH), 3.92 (d, *J* = 5.3 Hz, 2H, NCH_2_), 3.48 (s, 2H, ArCH_2_), 2.93 (s, 3H, CH_3_), 2.83 (s, 3H, CH_3_). LC-MS: *m*/*z* 397.1 [M + H]^+^, 395.2 [M − H]^−^.

### 3.3. Protease Inhibition Assay

In vitro activities of guanidinium drug analogues **1a**–**1e** were measured by a TMPRSS2 fluorogenic assay kit (CAT # 780837 BPS Bioscience, San Diego, CA, USA) according to the manufacturer’s recommendations.

Stock solutions of test compounds were prepared in DMSO (5% *v*/*v*), and 10 µL was transferred to the respective wells at a range of concentrations (0.007, 0.013, 0.026, 0.053 and 0.1054 µM; 1% *v*/*v* DMSO final). TMPRSS2 (30 µL, 5 ng/µL) was added to all wells, excluding the blank. Following incubation (30 min, room temperature), TMPRSS2 substrate (10 µL, 50 µM) was added and incubated at room temperature, protected from light. The fluorescence intensity was then measured in darkness by a multimode microplate reader (FLUOstar Omega, BMG Labtech, Allmendgrün, Ortenberg, Germany) at emission and excitation wavelengths of 383 nm and 455 nm, respectively. Camostat mesylate (10 µM) provided with the kit was used as the inhibitor control, according to Hoffmann et al. [15]. The positive control consisted of no inhibitor. Blank reactions without inhibitor or enzyme formed the negative control, and these background values were subtracted from all measurements. Enzyme activity was represented as the percentage of protease activity in the absence of the inhibitor, set at 100%. The data were analysed using GraphPad Prism 10 (GraphPad, Boston, MA, USA).

## 4. Conclusions

In this study, we investigated structural analogues of camostat, initially assessing all compounds via in silico docking studies, followed by in vitro evaluation of the top-binding candidates using fluorescence-based TMPRSS2 inhibition assays. Two of the analogues (**1c** and **1d**) exhibited inhibition potencies comparable to those of the parent drug, as confirmed by both the docking and inhibition methods. Our in vitro fluorescence-based protease assays, supported by computational docking studies, indicate that C-terminal camostat analogues retain TMPRSS2 inhibitory potency (IC_50_ = 1–3 nM; binding energy = –6.6 to –7.0 kcal/mol) comparable to or exceeding that of the parent compound.

This tolerance of structural variance points to a considerable scope for further fine-tuning of the drug’s C-terminal ‘tail’ in future compound libraries. The discrepancy between the strong binding seen in silico and the apparent lack of enzyme inhibition for the amide analogue (**1e**) offers evidence for the irreversible acylation mechanism of drug action. Acylation may be more thoroughly probed in camostat analogues demonstrating enhanced electrophilicity towards Ser441, for example, by incorporating electron-withdrawing (and sterically negligible) fluorination into the aromatic rings adjacent to the scissile ester.

The diminished binding computationally predicted for derivatives featuring flexible and extended linker units suggests that significant rigidity and conformational preorganisation are required for the effective binding of guanidinium-based drugs within the TMPRSS2 binding pocket. These promising results can collectively inform the design of further, more potent analogues of camostat and other guanidinium-based protease inhibitors. Through further such SAR cycles, we aim to advance their repurposing as antiviral therapies, particularly against coronavirus and other currently problematic respiratory viruses.

## Figures and Tables

**Table 1:** Predicted binding energies (BEs) for the complexes of the camostat analogues **1a** to **1d** within the catalytic site of protease TMPRSS2 (PDB ID: 7MEQ).

Compound	Top Pose (kcal/mol)	Top Three Poses (ave.) (kcal/mol)	st. dev. (kcal/mol)
**1a/b**	−6.6	−6.4	±0.2
**1c**	−7.0	−6.7	±0.3
**1d**	−7.0	−6.6	±0.3
**1e**	−6.8	−6.7	±0.1
**1f**	−6.0	−5.9	±0.2
**1g**	−5.7	−5.6	±0.1
**1h**	−6.3	−6.1	±0.3

**Table 2:** IC_50_ values from in vitro TMPRSS2 enzyme inhibition activity for camostat analogues **1a** to **1e**. IC_50_ stated ± SEM (standard error on the mean) Camostat was used as the control (10 µM) for maximum inhibition. The data shown are the average of two independent experiments, each performed with duplicate samples (N = 4). nd = not determined.

Compound	IC_50_ (nM)	SEM
**1a**	1.1	±0.1
**1b**	2.4	±0.2
**1c**	1.3	±0.1
**1d**	1.8	±0.1
**1e**	nd	-

## Data Availability

The original contributions presented in this study are included in the article and Supplementary Materials. Further inquiries can be directed to the corresponding authors.

## Supplementary Materials

The following supporting information can be downloaded at: https://www.mdpi.com/article/10.3390/ijms26146761/s1.

## Abbreviations

The following abbreviations are used in this manuscript:

BE	Binding energy
BOC	*tert*-Butyloxycarbonyl
DCM	Dichloromethane
DIPEA	*N*,*N*-Diisopropylethylamine
DMAP	4-(Dimethylamino)pyridine
DMF	*N*,*N*-Dimethylformamide
DMSO	Dimethyl sulfoxide
EDCI	1-Ethyl-3-(3-dimethylaminopropyl)carbodiimide hydrochloride
GBPA	4-(Guanidinobenzoyloxy)phenylacetic acid
SAR	Structure–activity relationship
SEM	Standard error on the mean
TFA	Trifluoroacetic acid/trifluoroacetate
THF	Tetrahydrofuran
TLC	Thin-layer chromatography
TMPRSS2	Transmembrane serine protease 2
