# Supplementary material for: C-Terminal Analogues of Camostat Retain TMPRSS2 Protease Inhibition: New Synthetic Directions for Antiviral Repurposing of Guanidinium-Based Drugs in Respiratory Infections
Source: Int J Mol Sci. 2025 Jul 15;26(14):6761. doi: 10.3390/ijms26146761 (PMC12295440; doi:10.3390/ijms26146761)

Supporting Information

C-terminal analogues of camostat retain TMPRSS2 protease in-hibition: new synthetic directions for antiviral repurposing of guanidinium-based drugs in respiratory infections

Bill T. Ferrara[1], Elinor P. Thompson[1], Giovanni N. Roviello[2]* and Thomas F. Gale[1]*

[1] School of Science, Faculty of Engineering and Science, University of Greenwich, Central Avenue, Chatham Maritime, Kent ME4 4TB, United Kingdom

[2] CNR Institute of Biostructures and Bioimaging, Via Tommaso De Amicis 95, 80145, Naples, Italy * Correspondence: giovanni.roviello@cnr.it , t.f.gale@gre.ac.uk

Fig S1. ^{1}H NMR and LC MS characterization of **1b**

(^{1}H-NMR in DMSO-d_6)

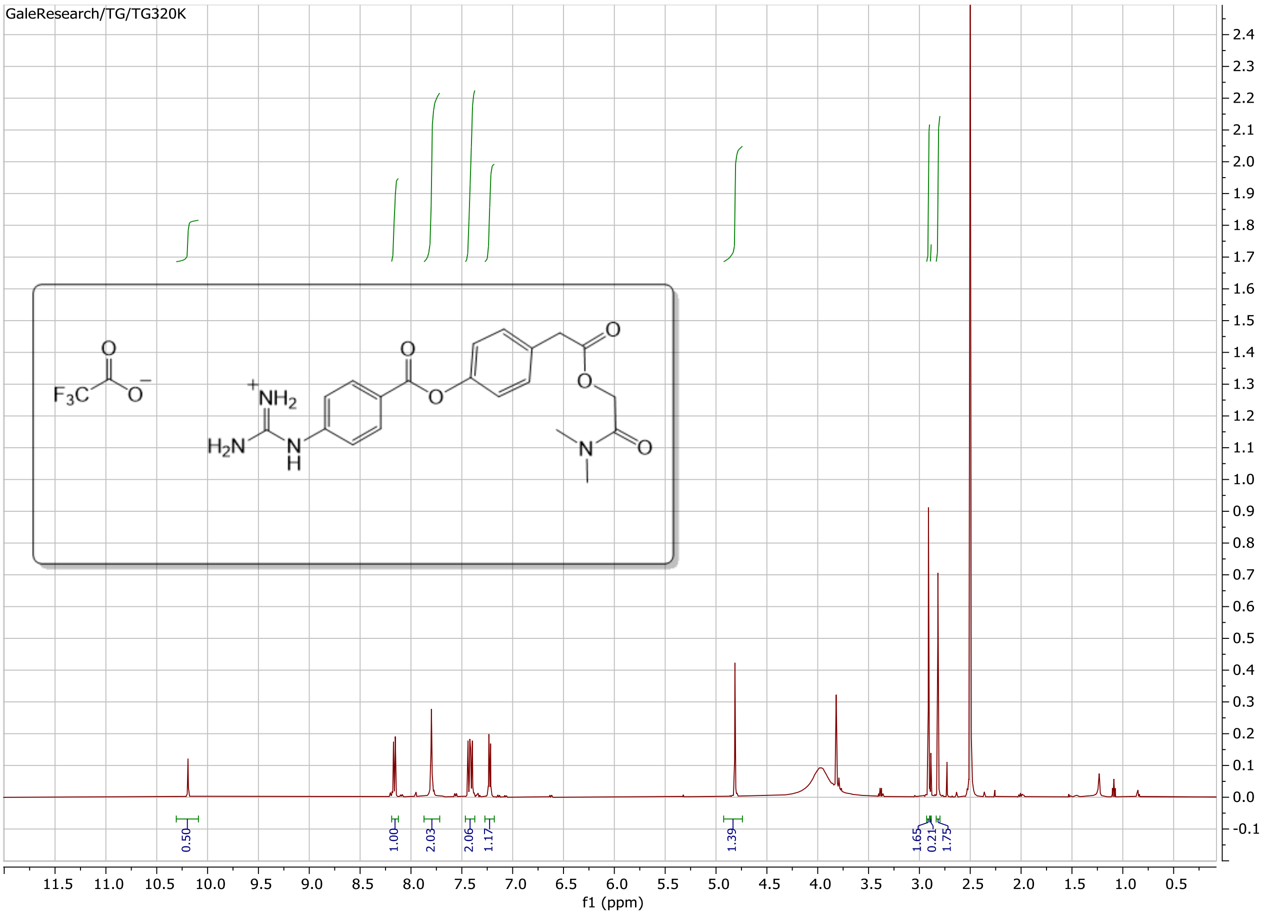

(^{1}LC-MS, reversed phase, H_2O-MeCN)

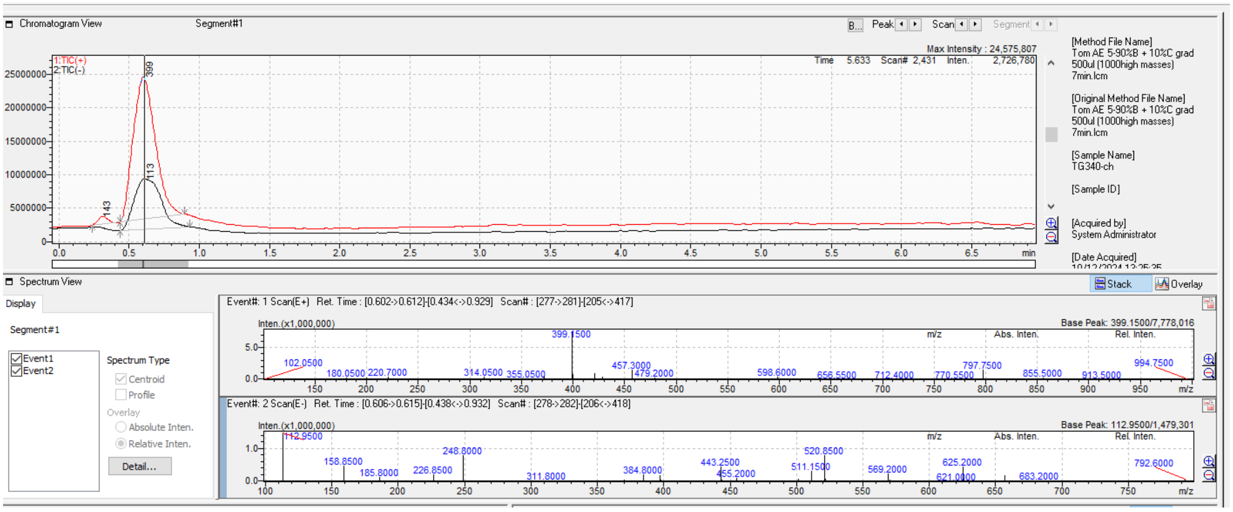

Fig S2. ^{1}H NMR and LC MS characterization of **1c**

(^{1}H-NMR in DMSO-d$_6$)

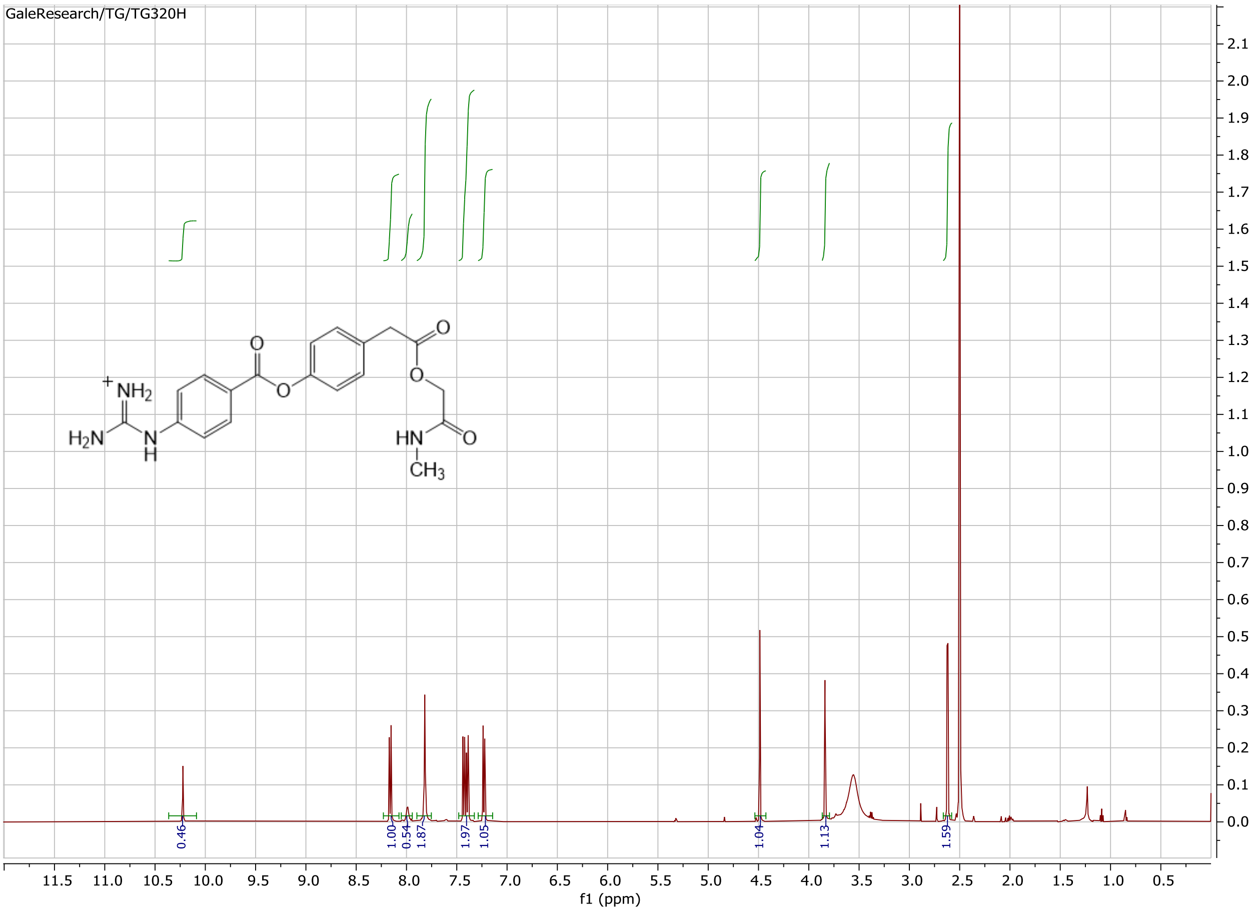

(^{1}LC-MS, reversed phase, H_2O-MeCN)

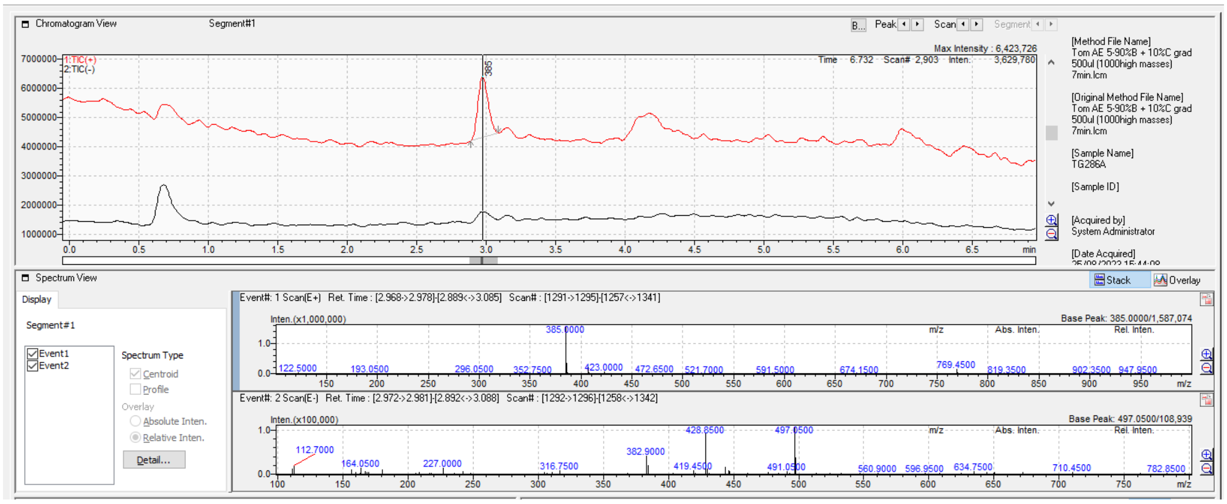

Fig S3. ^{1}H NMR and LC MS characterization of **1d**

(^{1}H-NMR in DMSO-d_6)

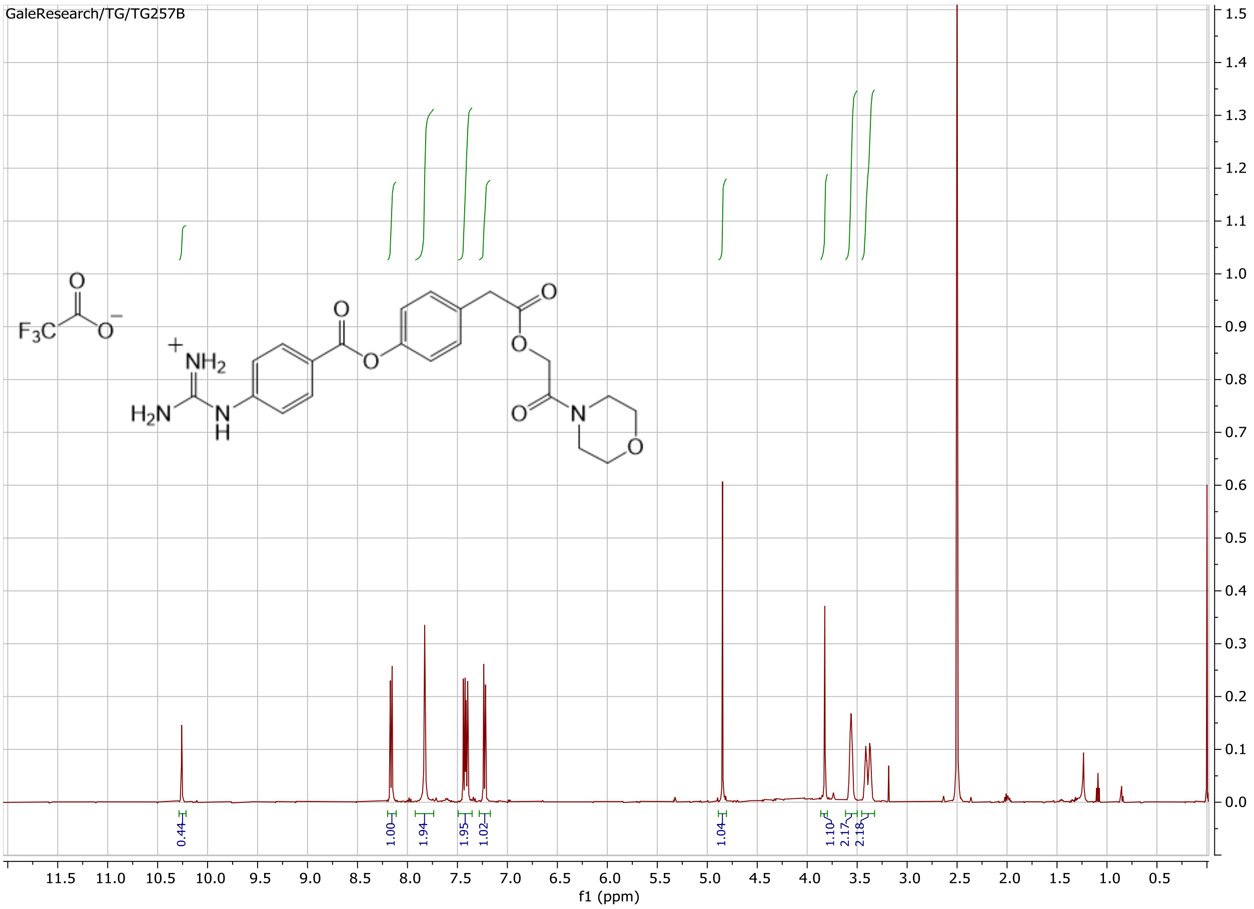

(^{1}LC-MS, reversed phase, H_2O-MeCN)

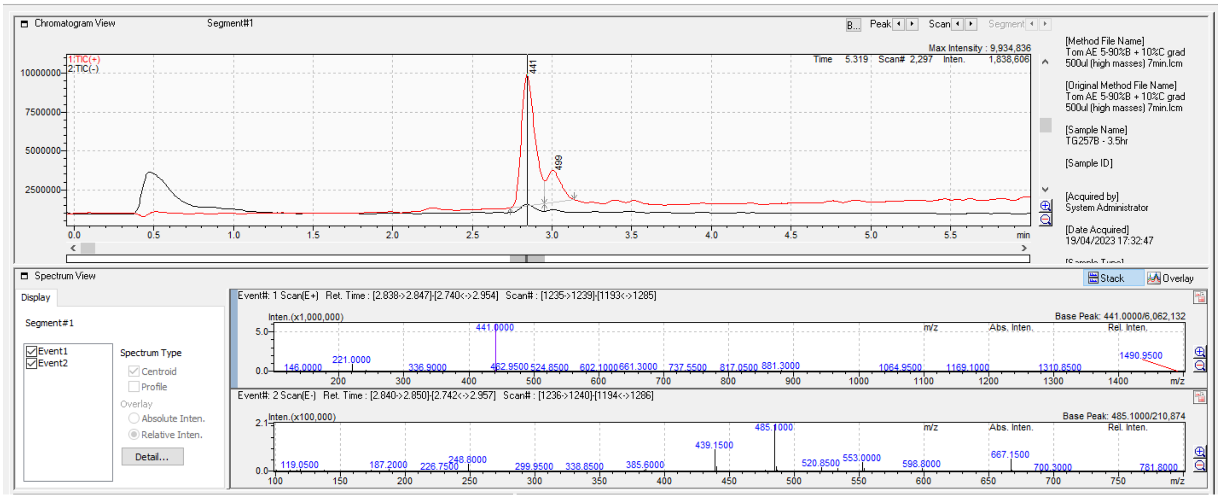

Fig S4. ^{1}H NMR and LC MS characterization of **1e**

(^{1}H-NMR in DMSO-d$_6$)

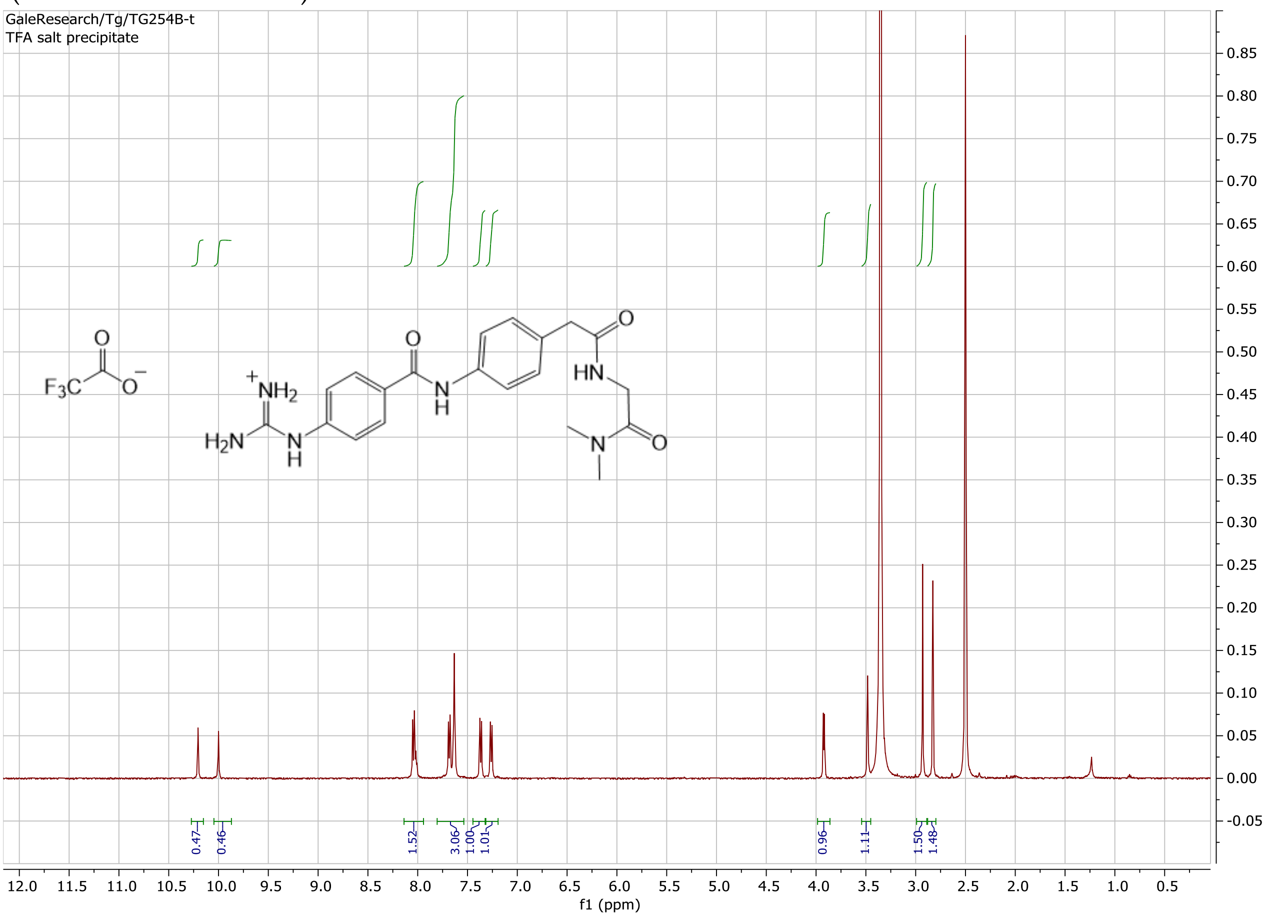

(^{1}LC-MS, reversed phase, H_2O-MeCN)

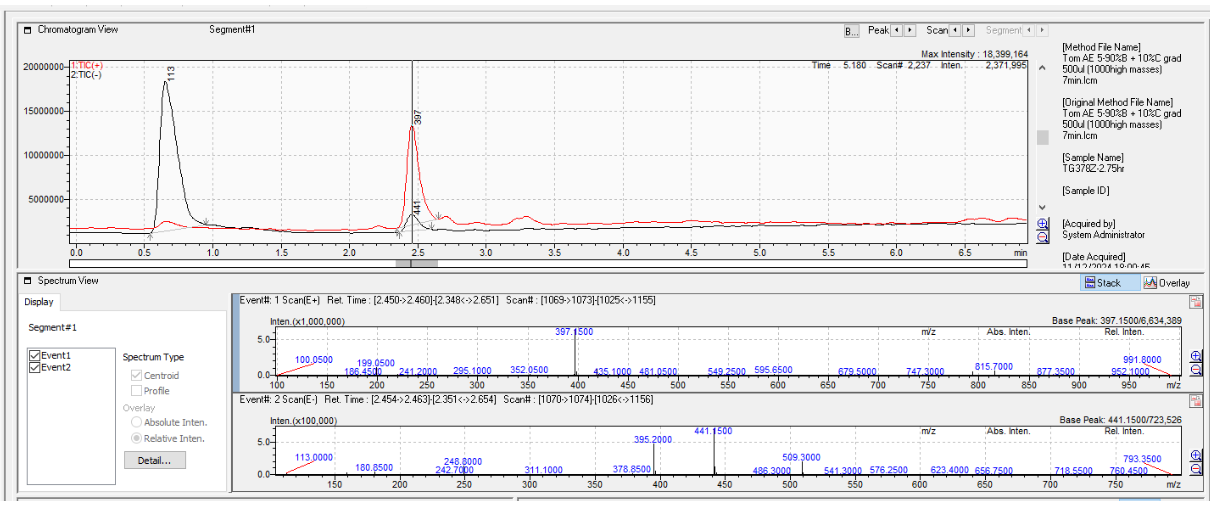

Fig S5. ^{1}H NMR and LC MS characterization of **4b**

(^{1}H-NMR in $CDCl_3$)

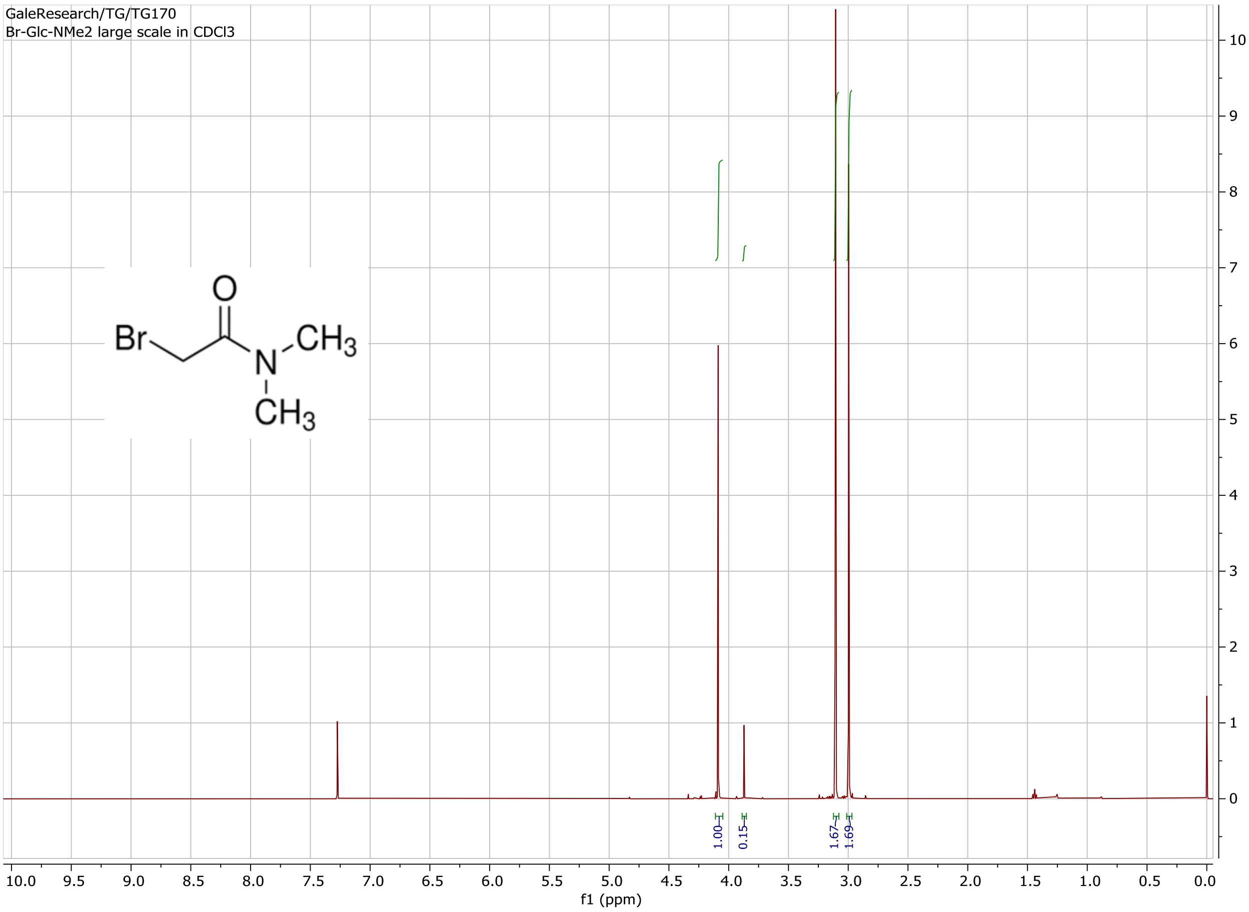

(^{1}LC-MS, reversed phase, H_2O-MeCN)

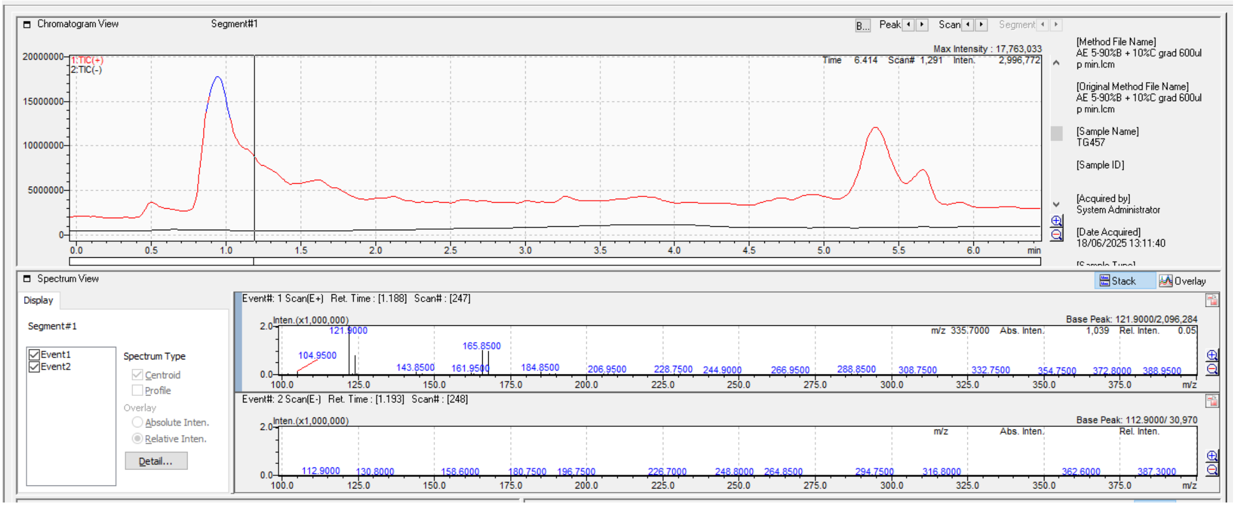

Fig S6. ^{1}H NMR and LC MS characterization of **4c**

(^{1}H-NMR in DMSO-d$_6$)

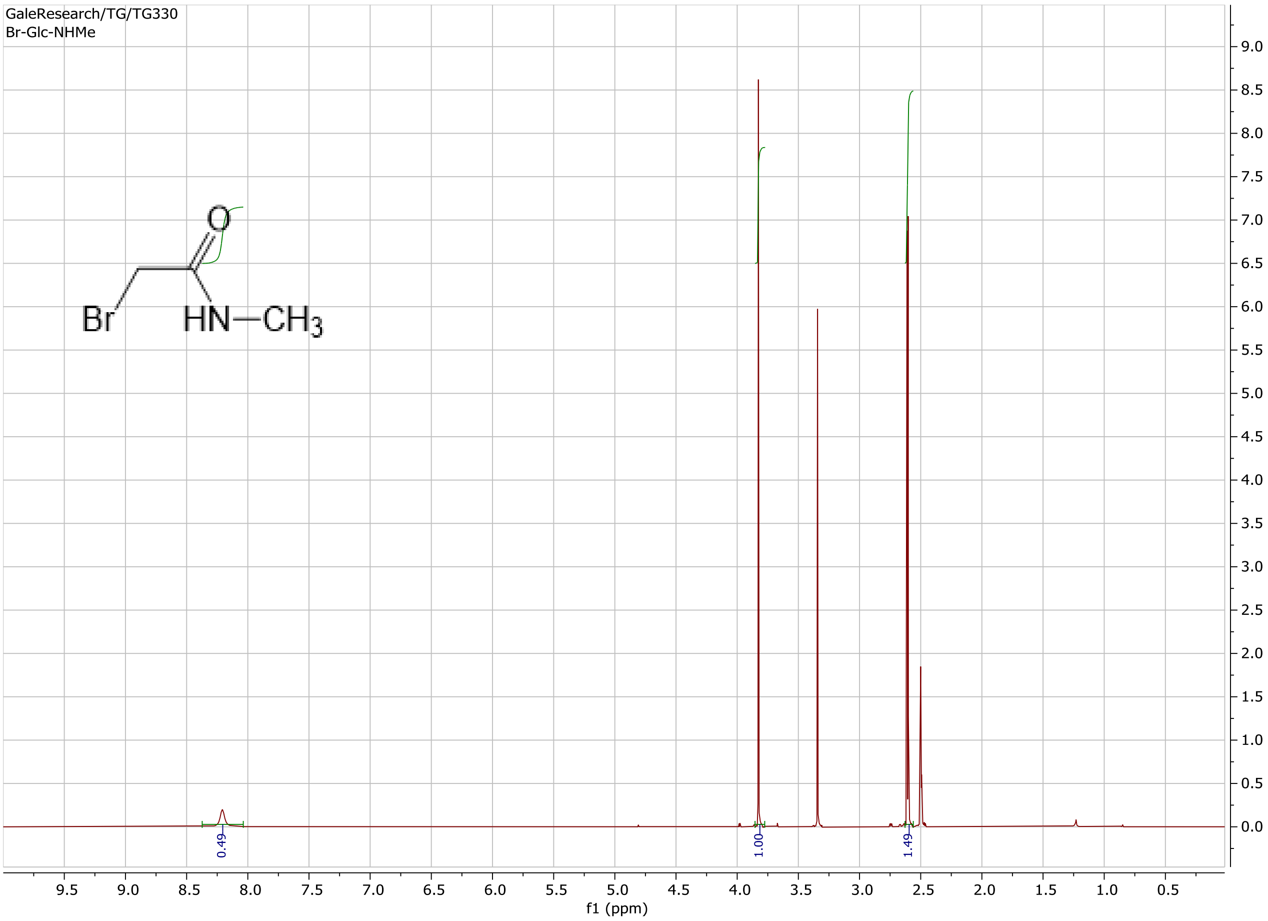

(^{1}LC-MS, reversed phase, H_2O-MeCN)

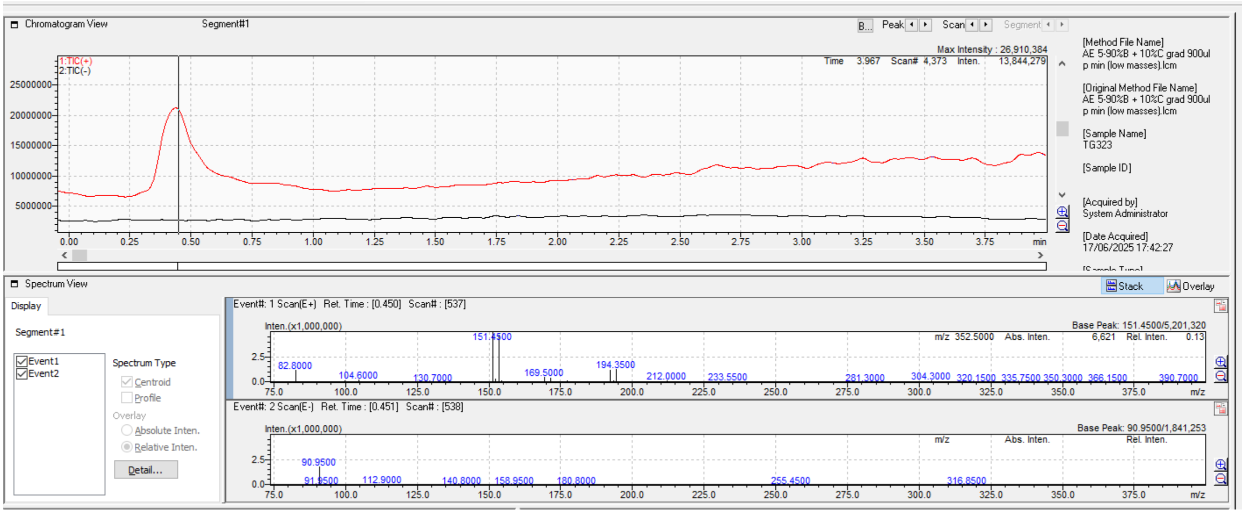

Fig S7. ^{1}H NMR and LC MS characterization of **4d**

(^{1}H-NMR in $CDCl_3$)

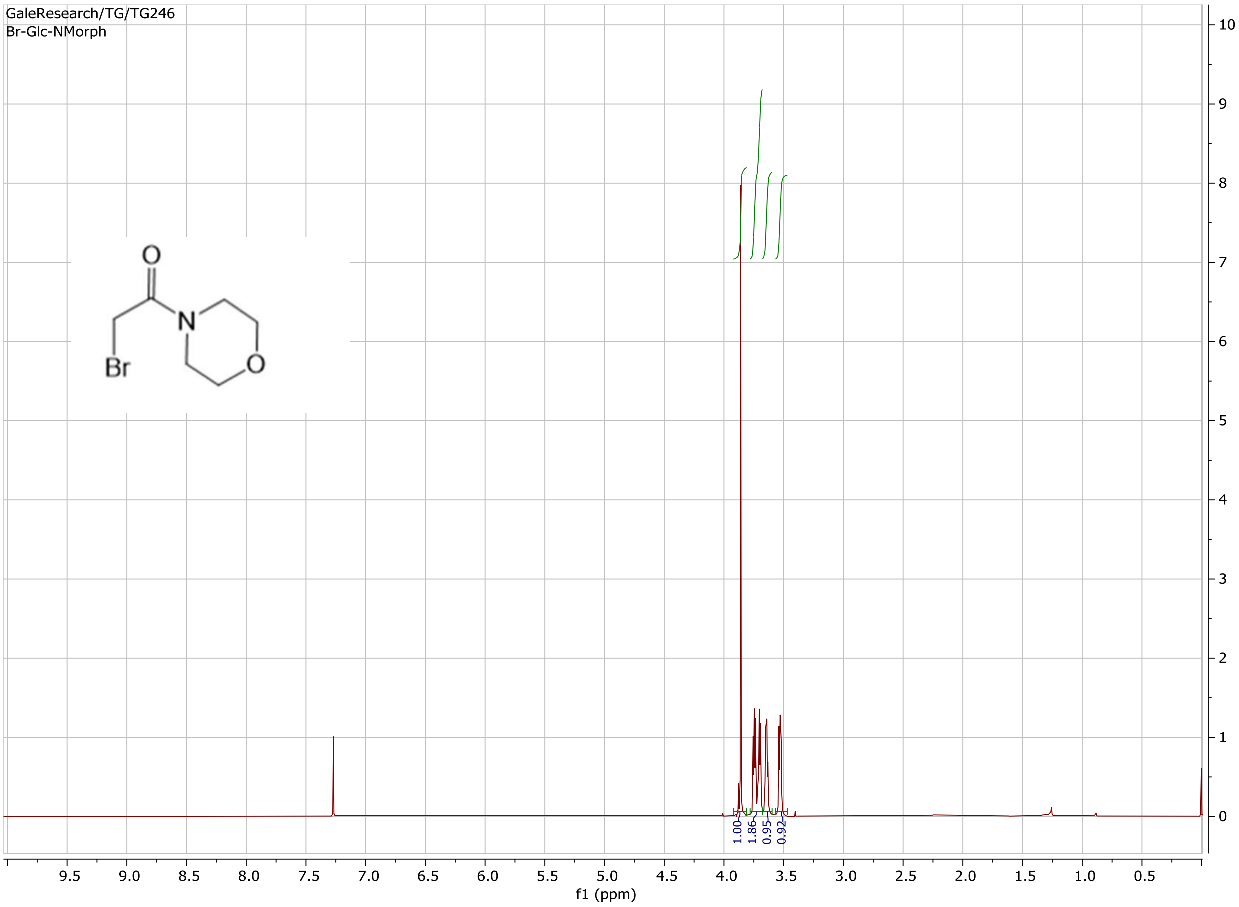

(^{1}LC-MS, reversed phase, H_2O-MeCN)

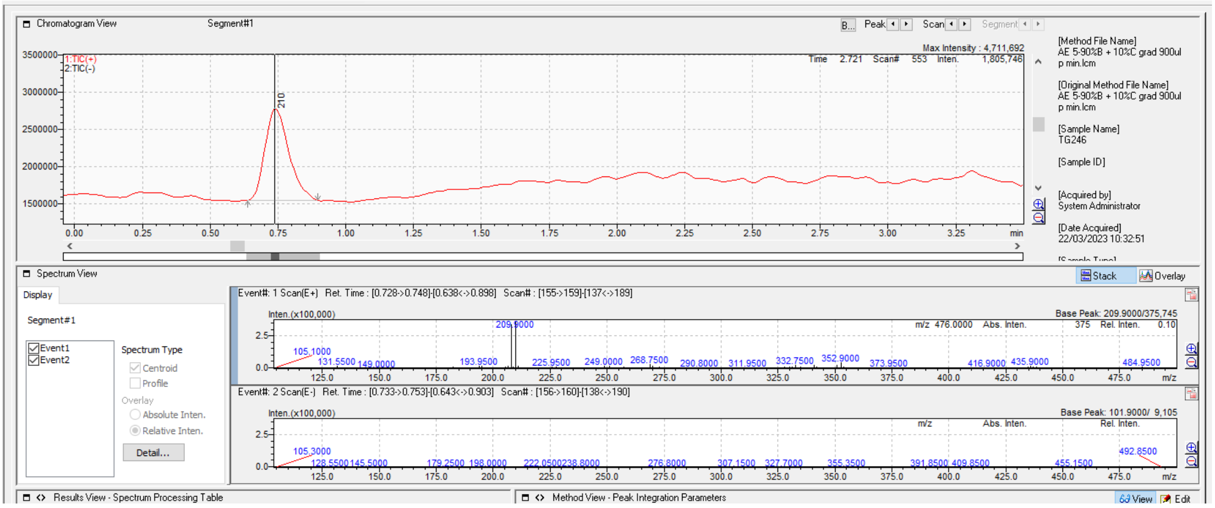

Fig S8. ^{1}H NMR and LC MS characterization of **5b**

(^{1}H-NMR in DMSO-d$_6$)

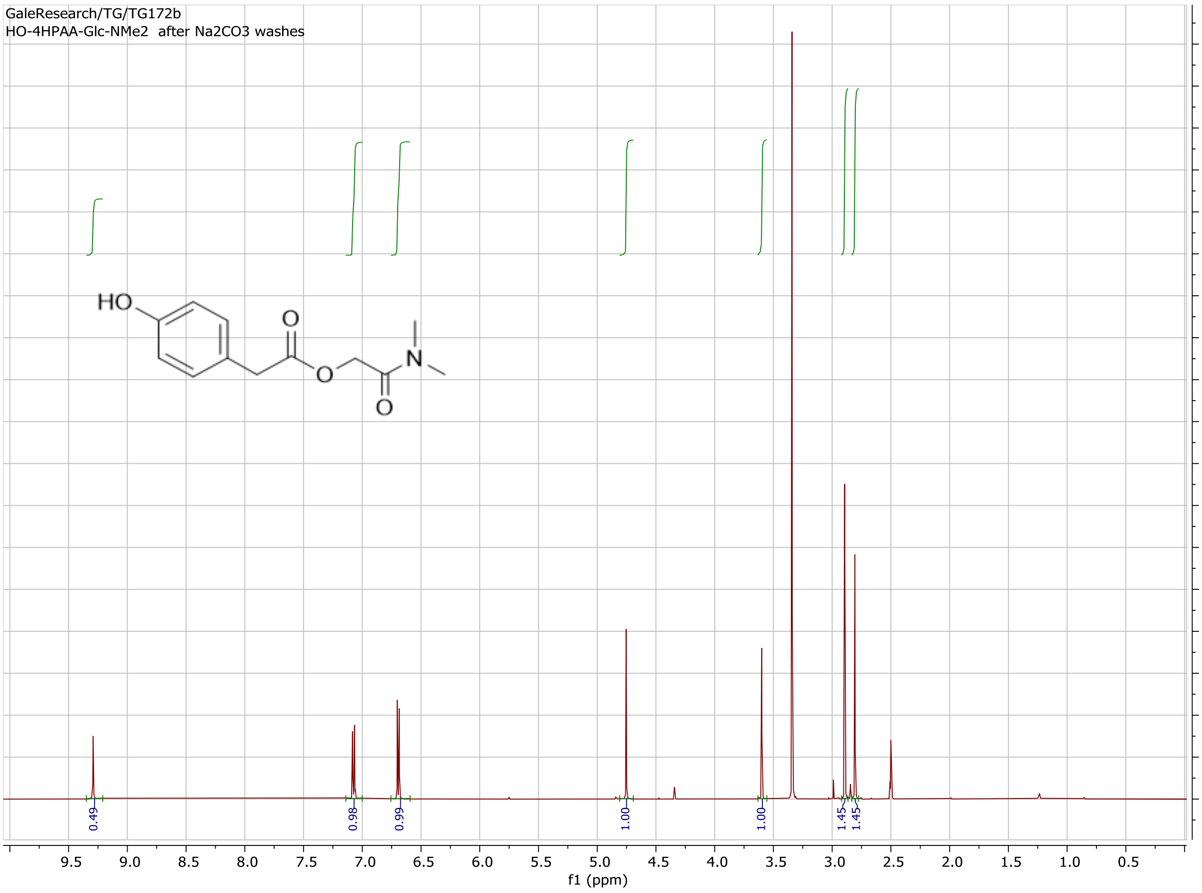

(^{1}LC-MS, reversed phase, H_2O-MeCN)

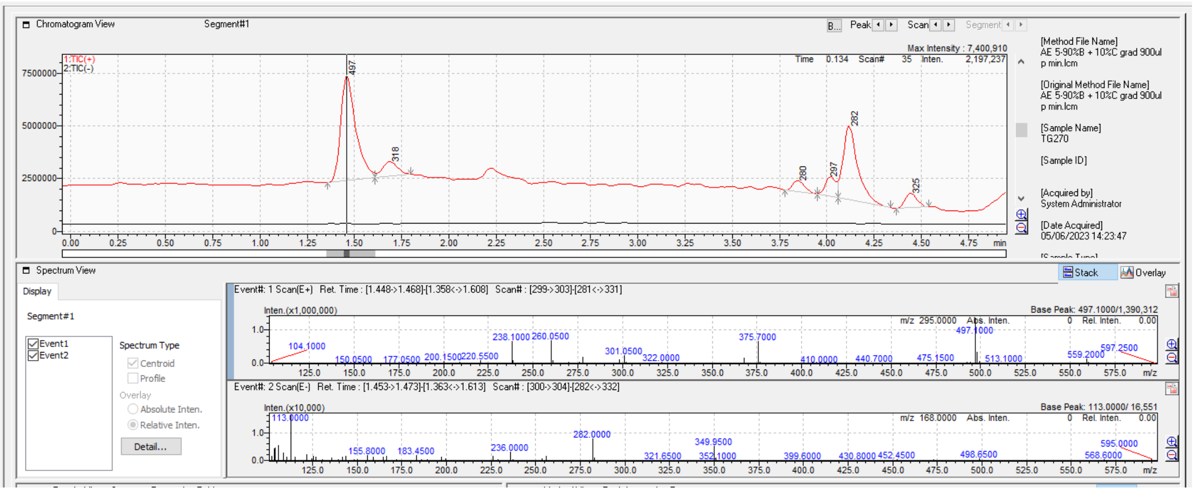

Fig S9. ^{1}H NMR and LC MS characterization of **5c**

(^{1}H-NMR in DMSO-d$_6$)

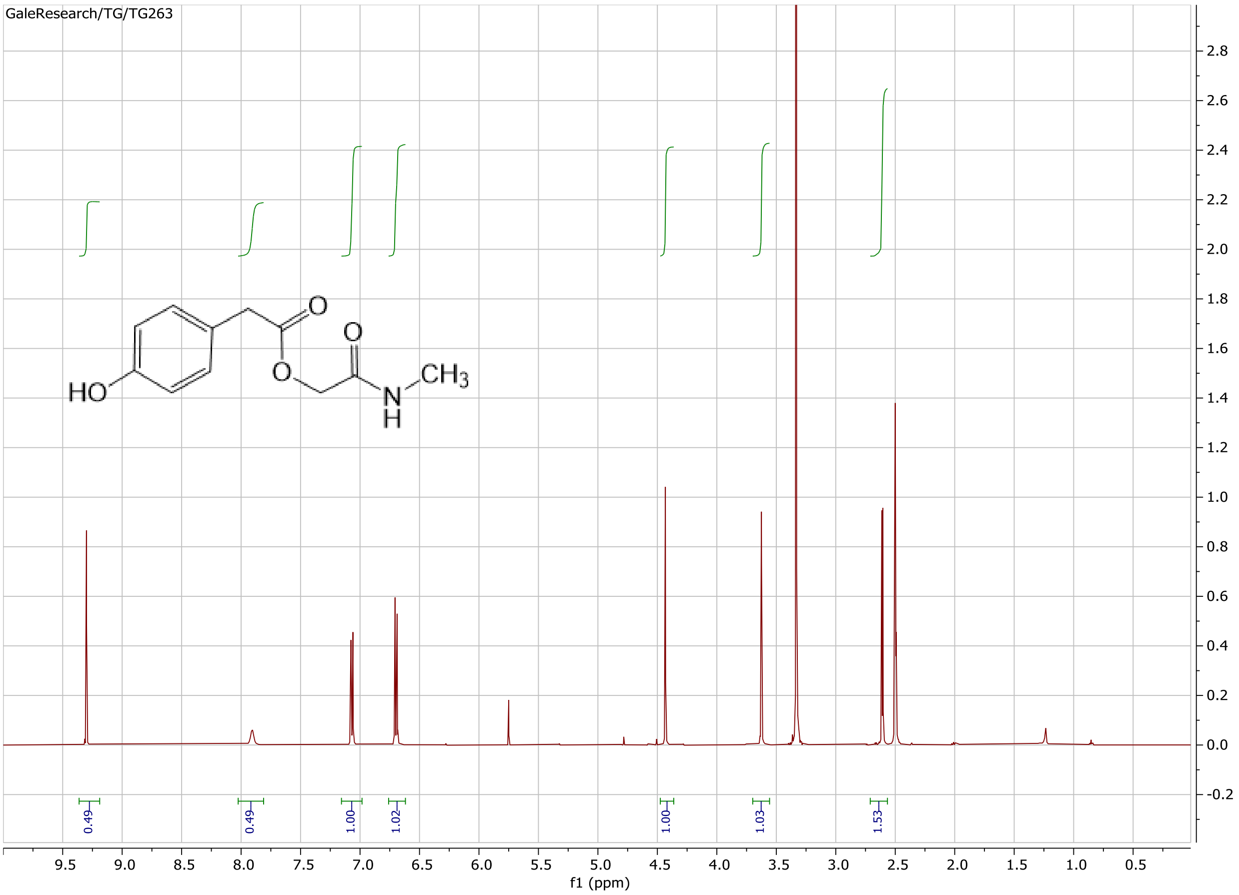

(^{1}LC-MS, reversed phase, H_2O-MeCN)

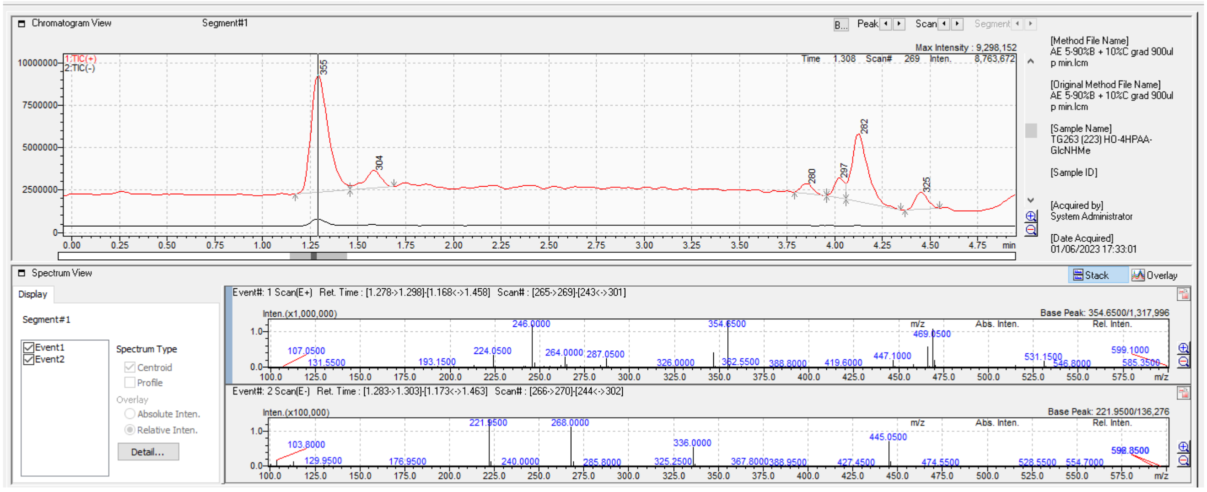

Fig S10. ^{1}H NMR and LC MS characterization of **5d**

(^{1}H-NMR in DMSO-d_6)

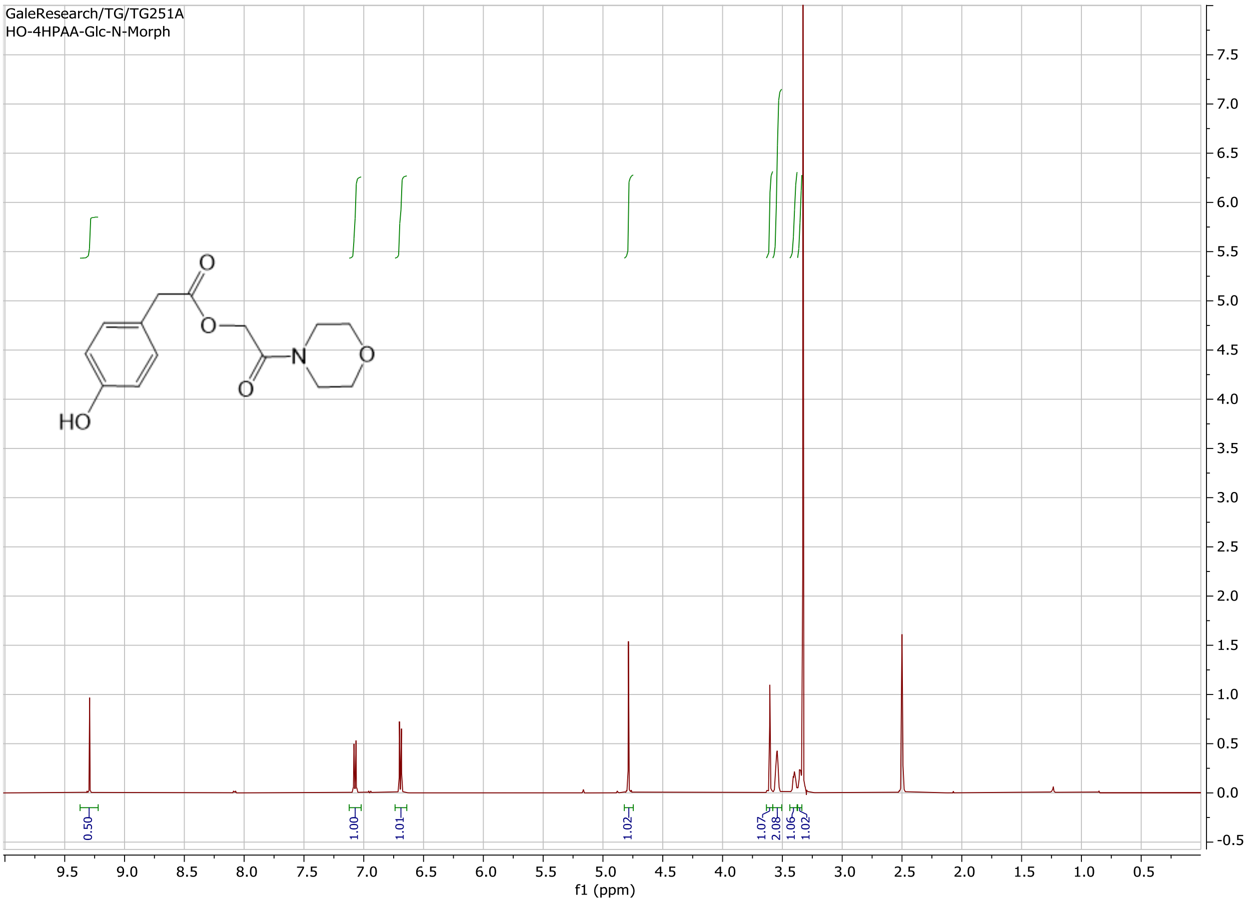

(^{1}LC-MS, reversed phase, H_2O-MeCN)

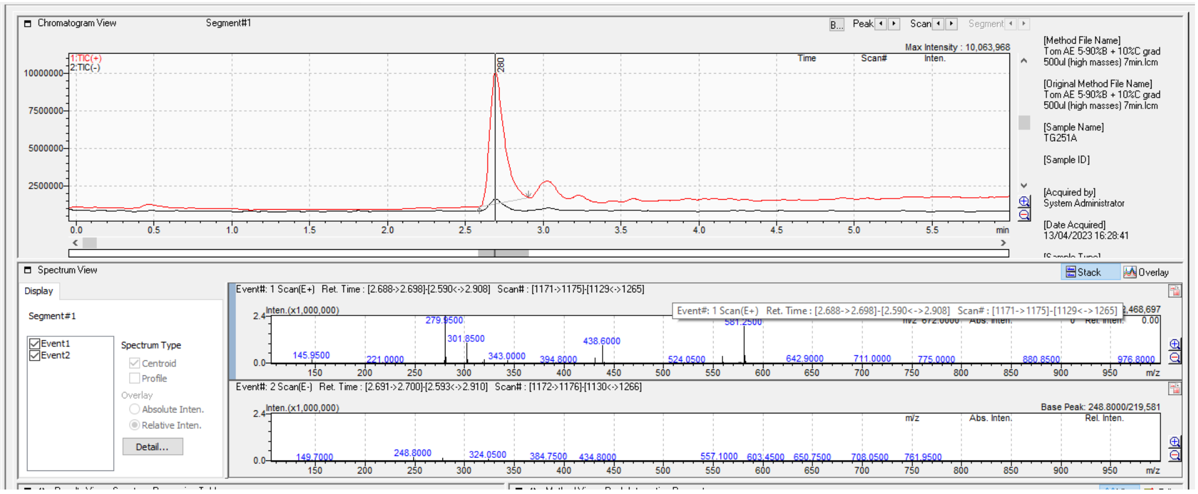

Fig S11. ^{1}H NMR and LC MS characterization of **7**

(^{1}H-NMR in DMSO-d$_6$)

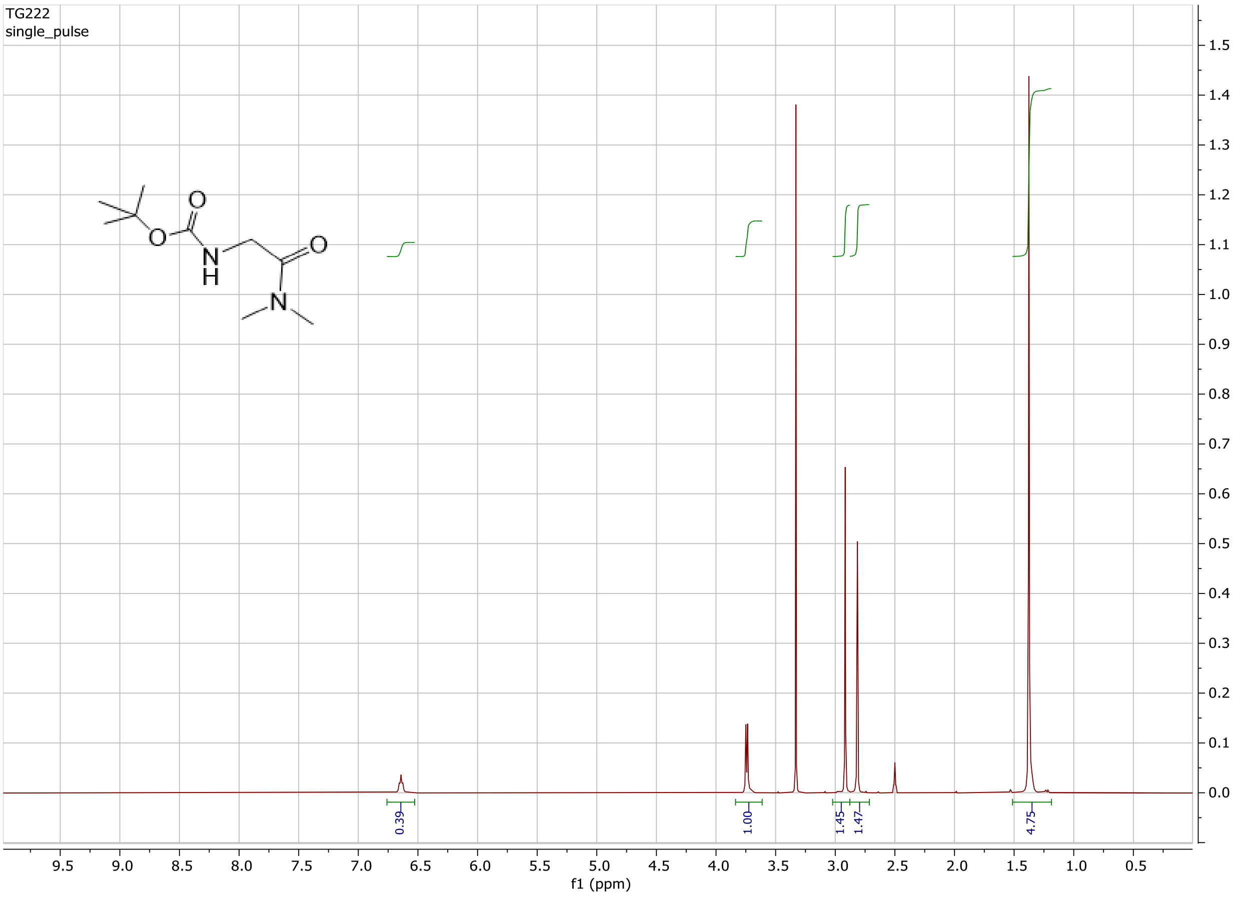

(^{1}LC-MS, reversed phase, H_2O-MeCN)

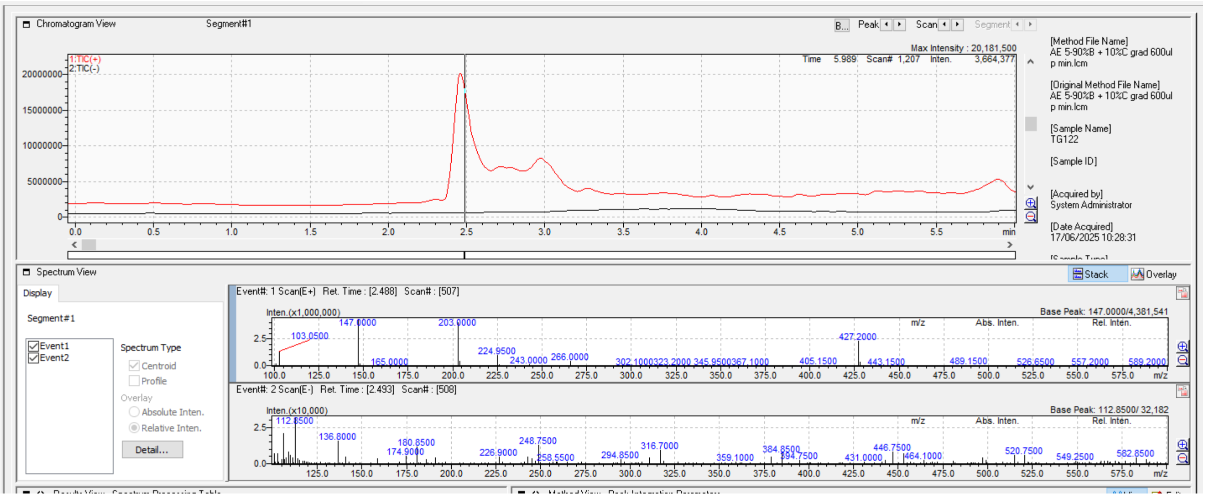

Fig S12. ^{1}H NMR and LC MS characterization of **8**

(^{1}H-NMR in DMSO-d_6)

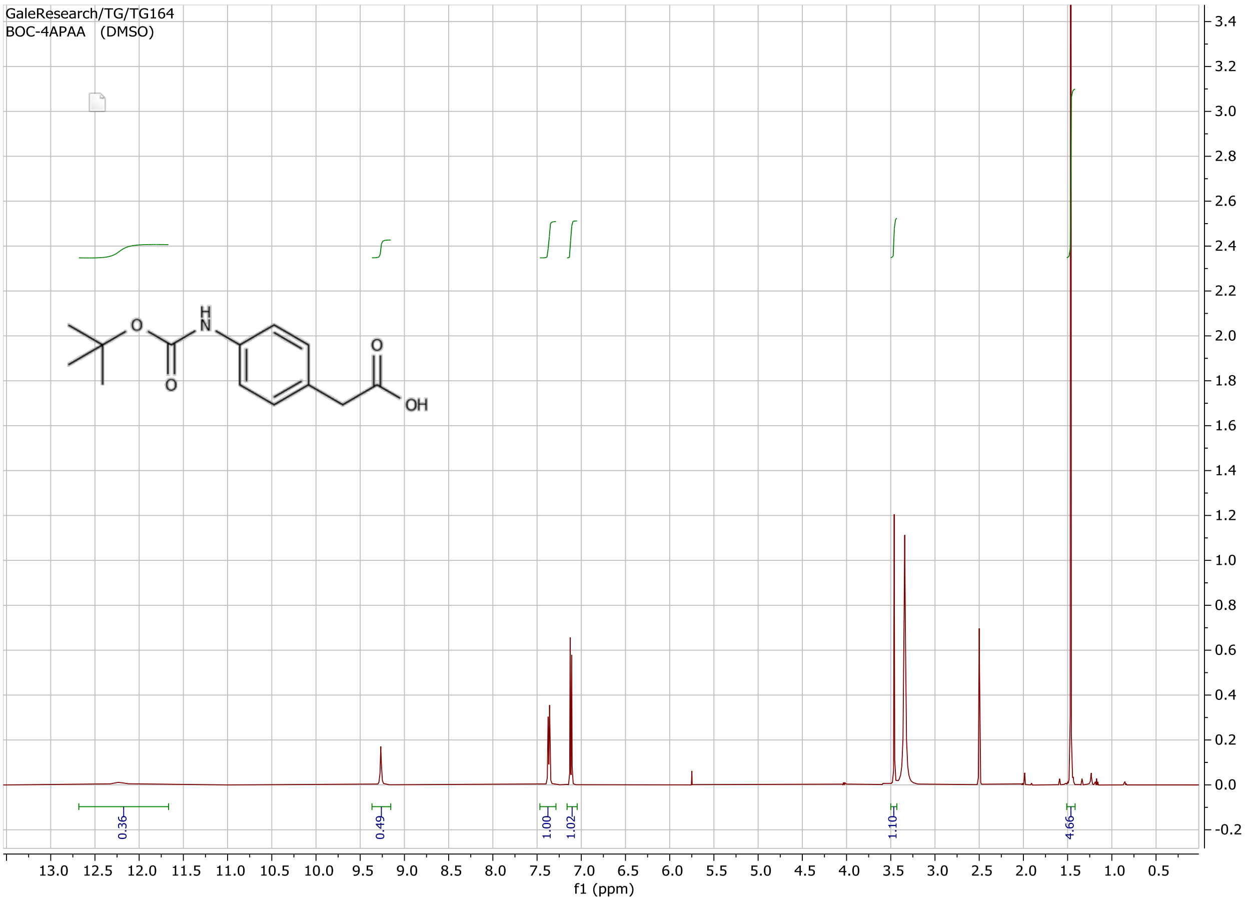

(^{1}LC-MS, reversed phase, H_2O-MeCN)

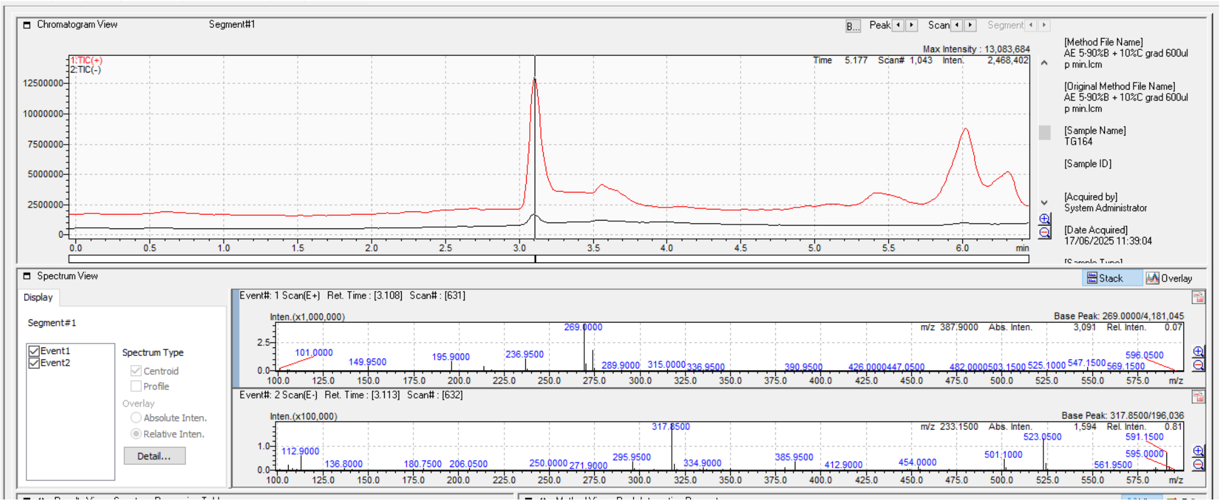

Fig S13. ^{1}H NMR and LC MS characterization of **9a**

(^{1}H-NMR in DMSO-d$_6$)

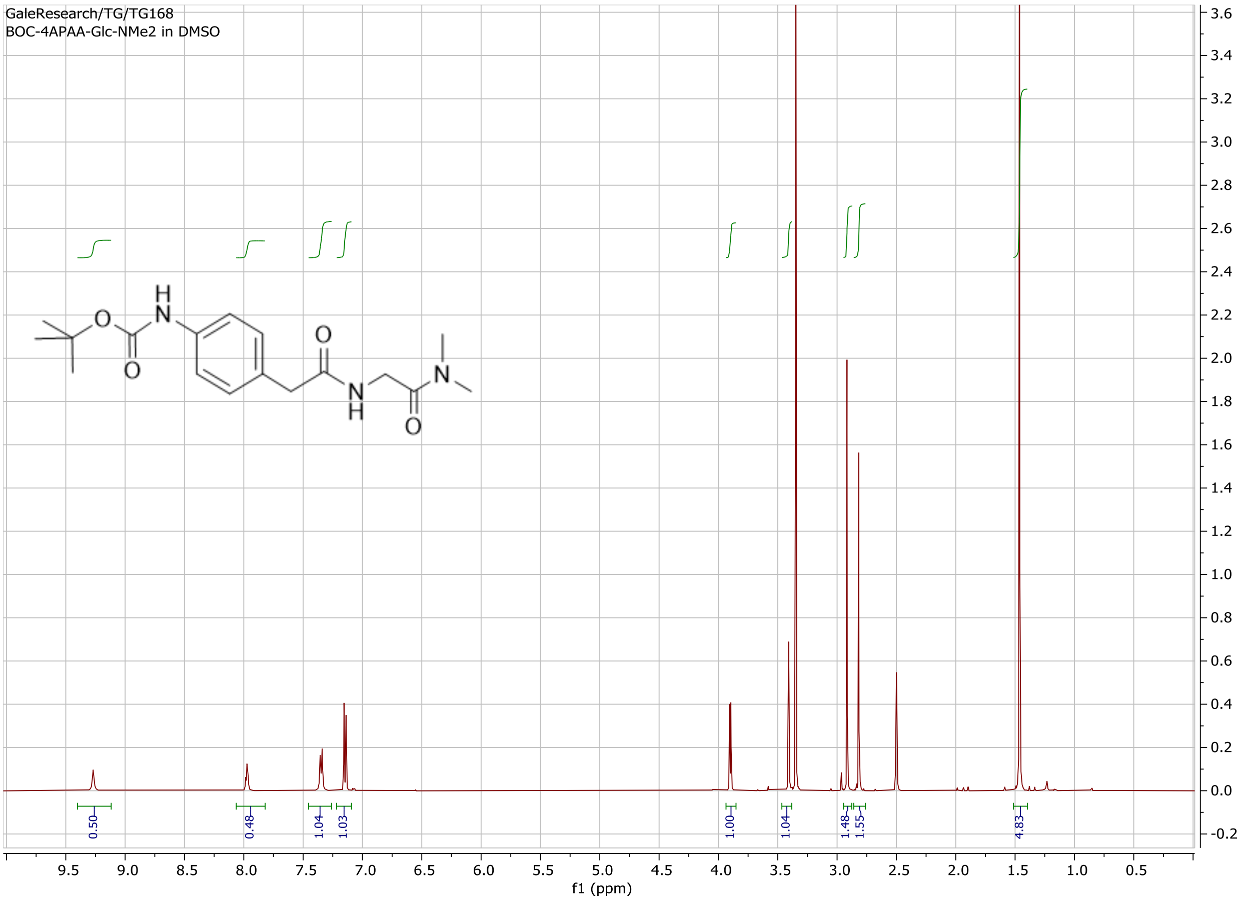

(^{1}LC-MS, reversed phase, H_2O-MeCN)

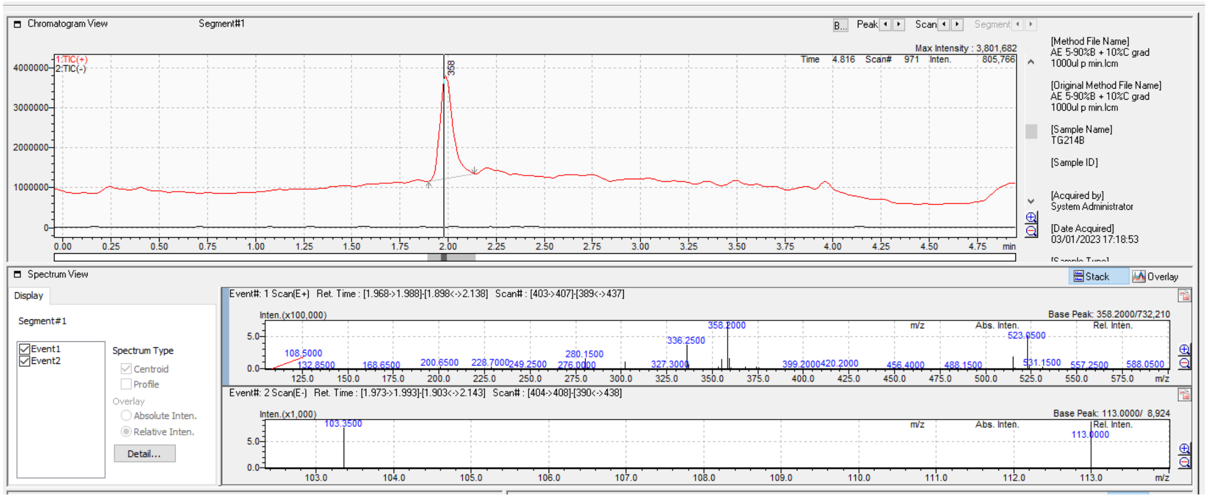

Fig S14. ^{1}H NMR and LC MS characterization of **11** (^{1}H-NMR in DMSO-d6)

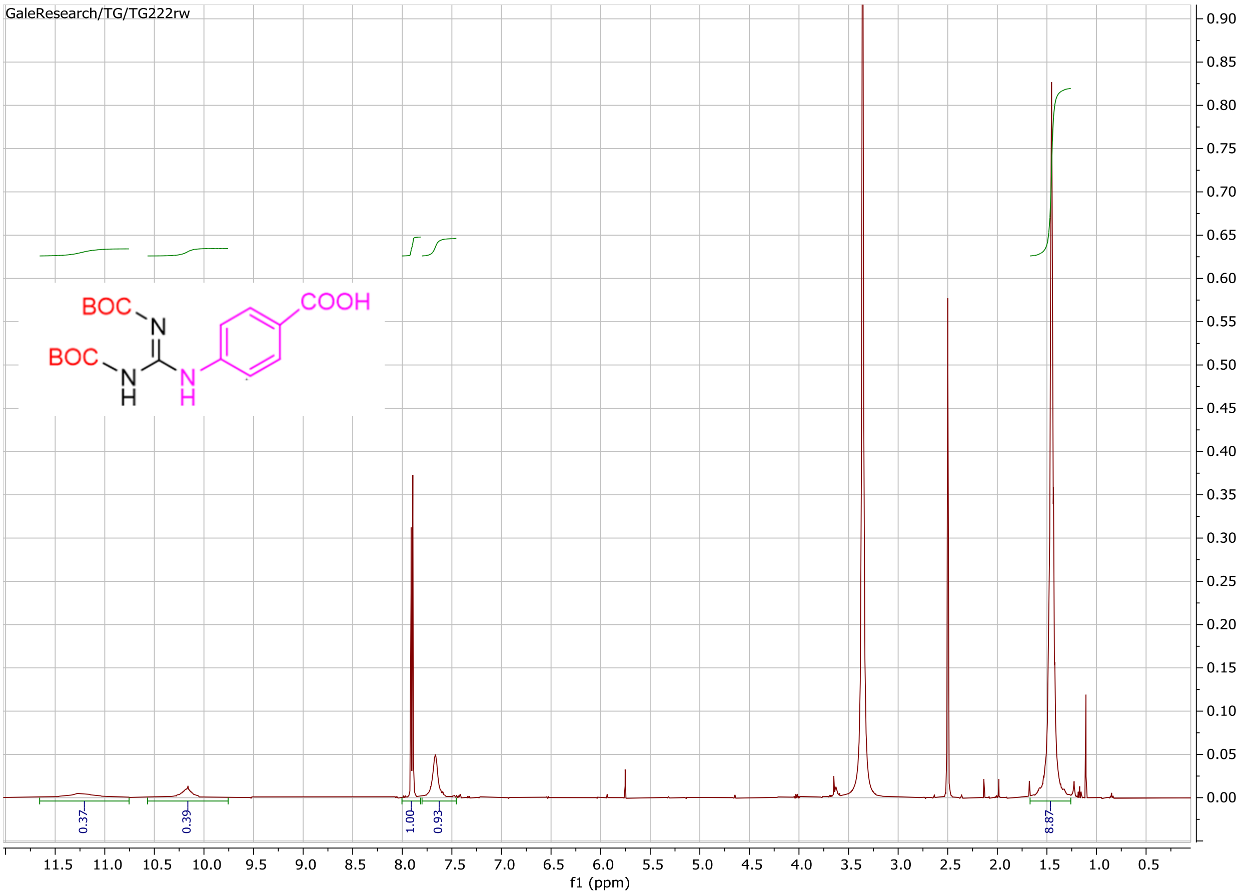

([1]LC-MS, reversed phase, H_2O-MeCN)

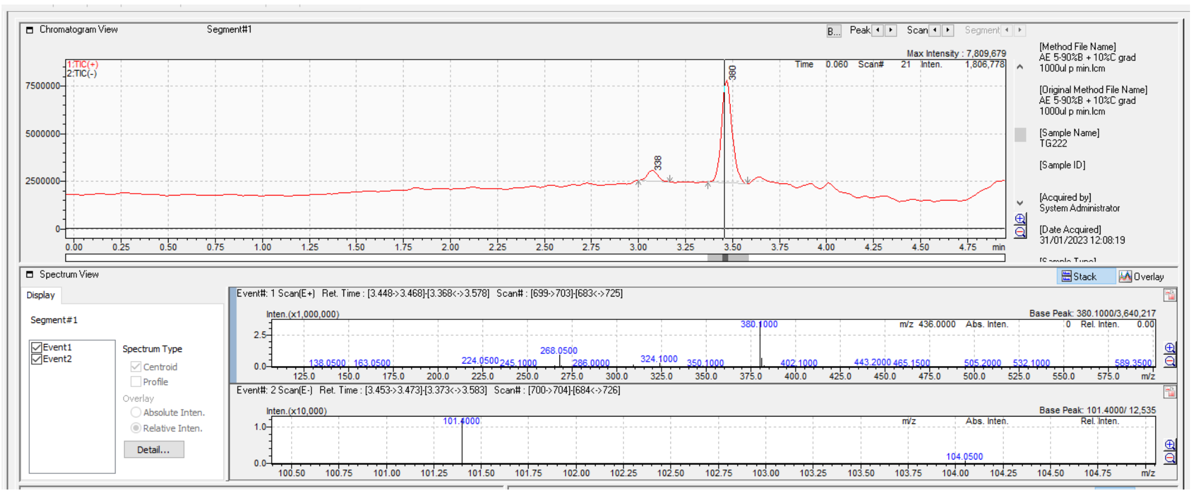

Fig S15. ^{1}H NMR and LC MS characterization of **12b**

(^{1}H-NMR in DMSO-d$_6$)

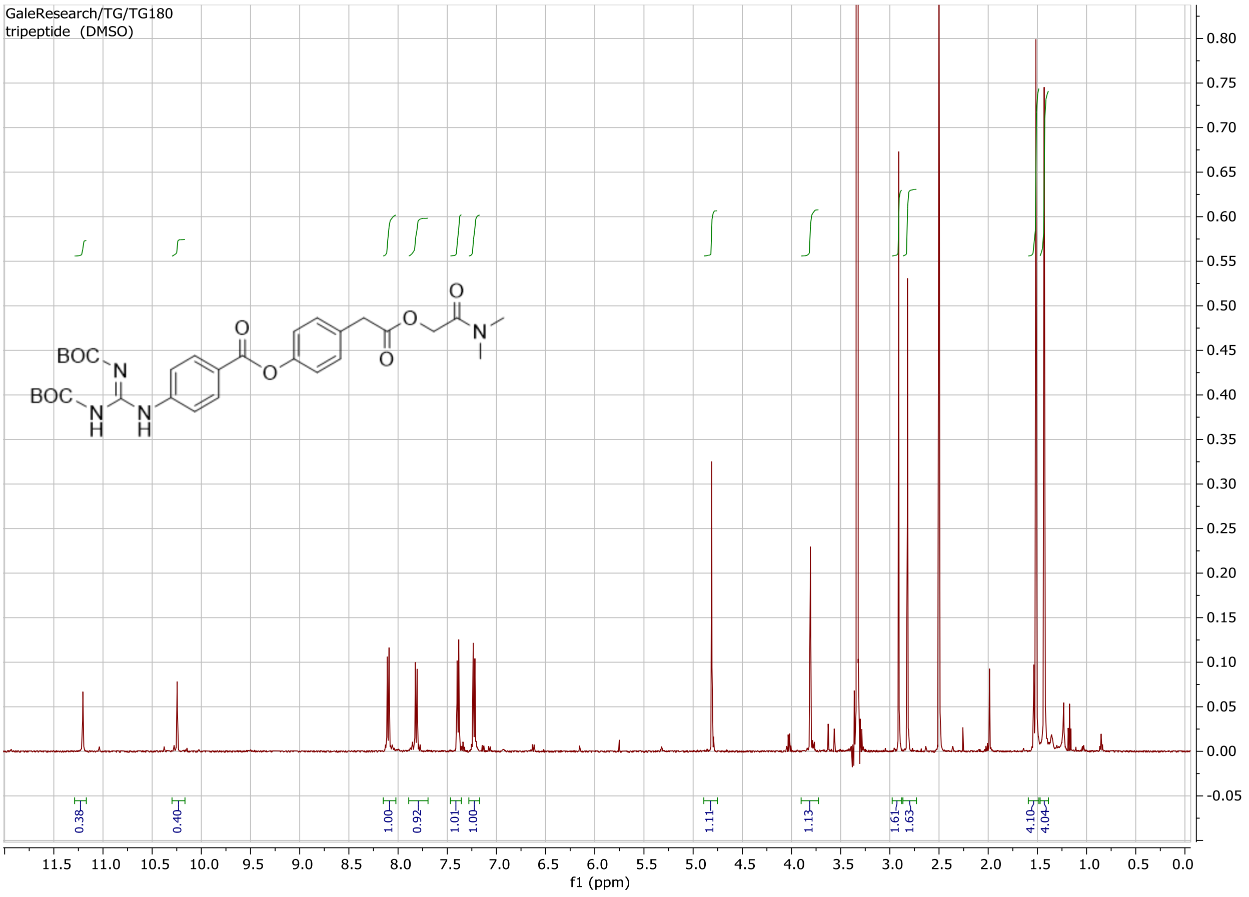

(^{1}LC-MS, reversed phase, H_2O-MeCN)

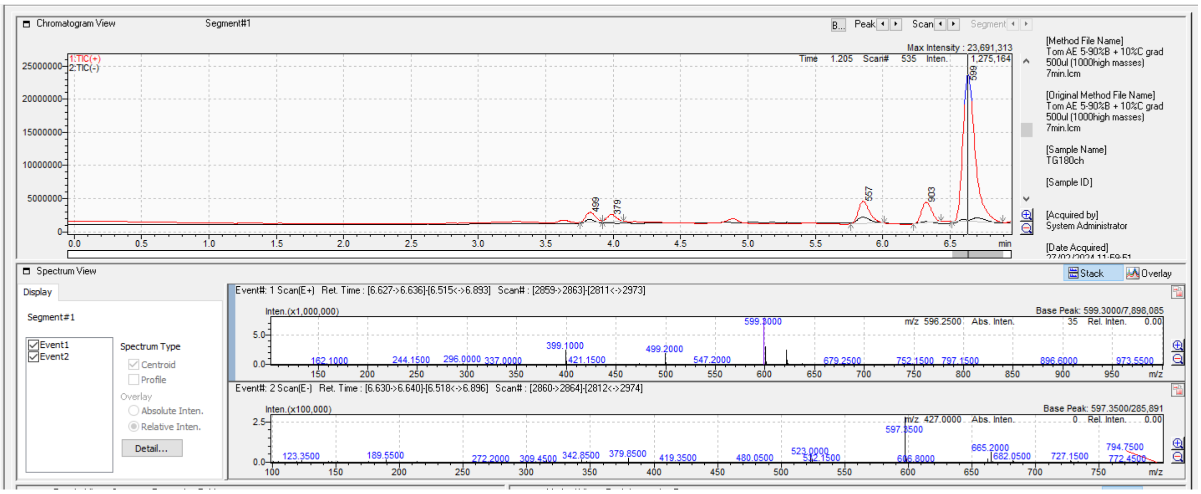

Fig S16. ^{1}H NMR and LC MS characterization of **12c**

(^{1}H-NMR in DMSO-d_6)

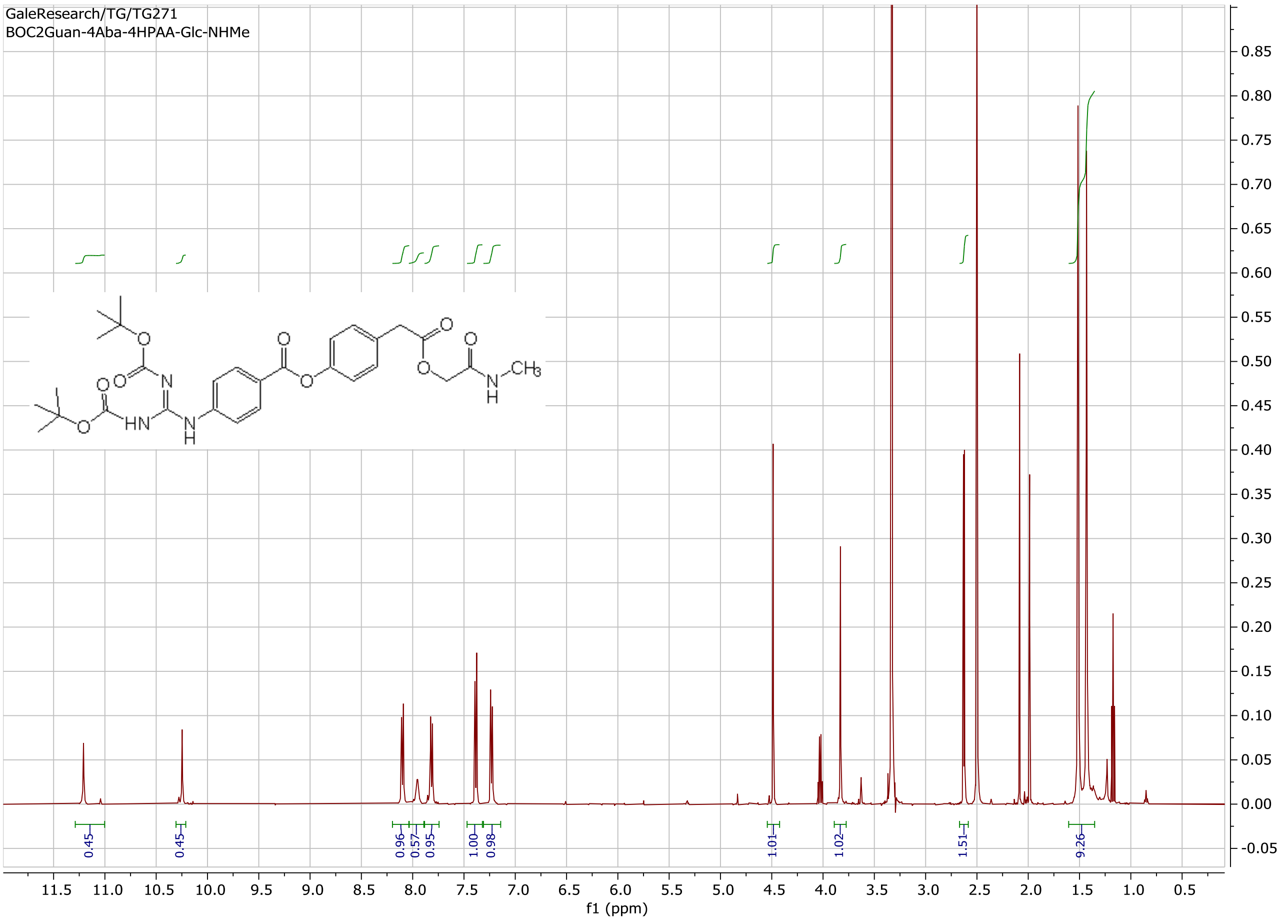

(^{1}LC-MS, reversed phase, H_2O-MeCN)

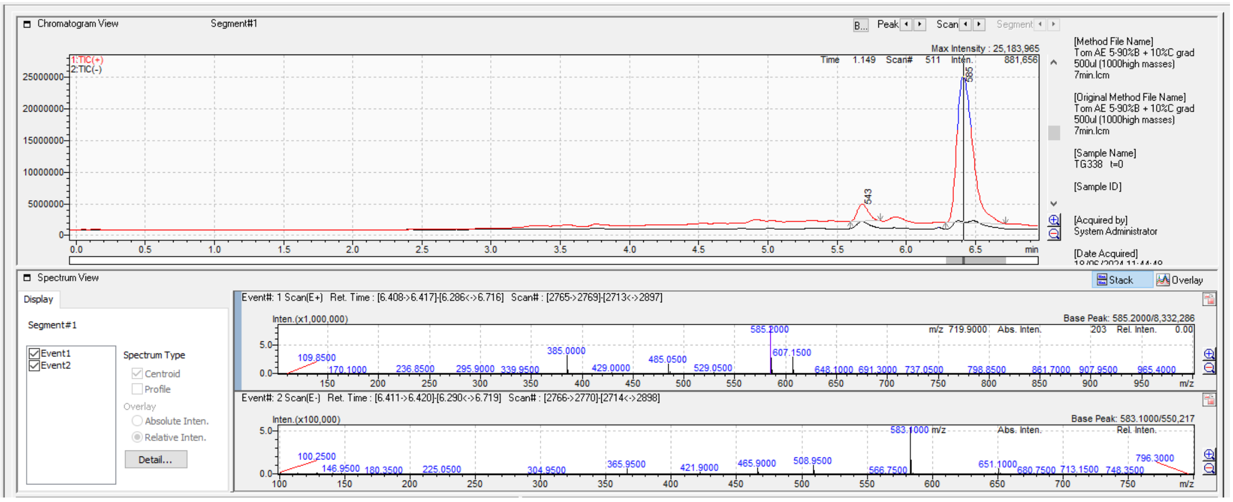

Fig S17. ^{1}H NMR and LC MS characterization of **12d**

(^{1}H-NMR in DMSO-d$_6$)

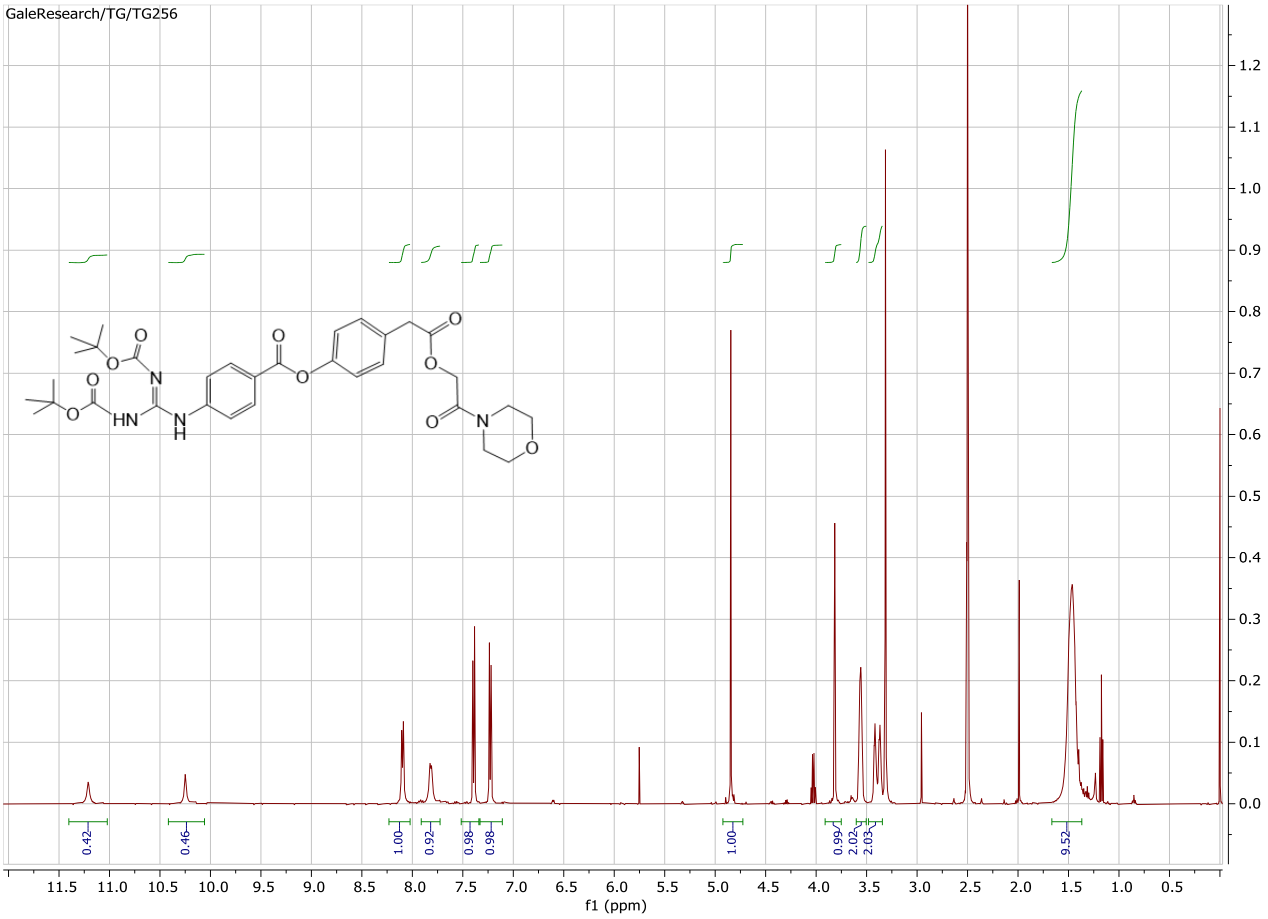

(^{1}LC-MS, reversed phase, H_2O-MeCN)

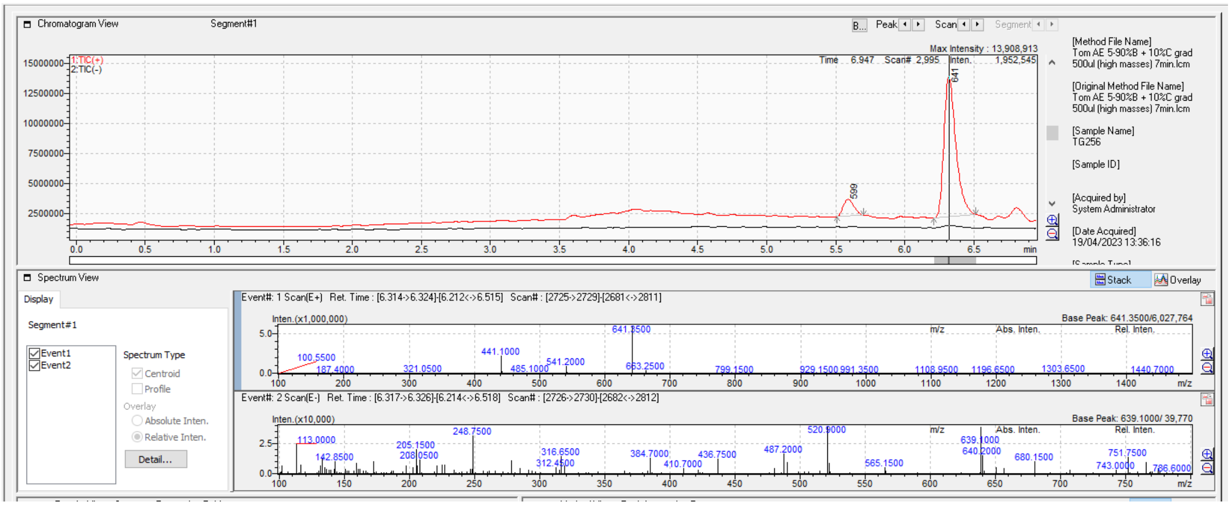

Fig S18. ^{1}H NMR and LC MS characterization of **12e**

(^{1}H-NMR in DMSO-d_6)

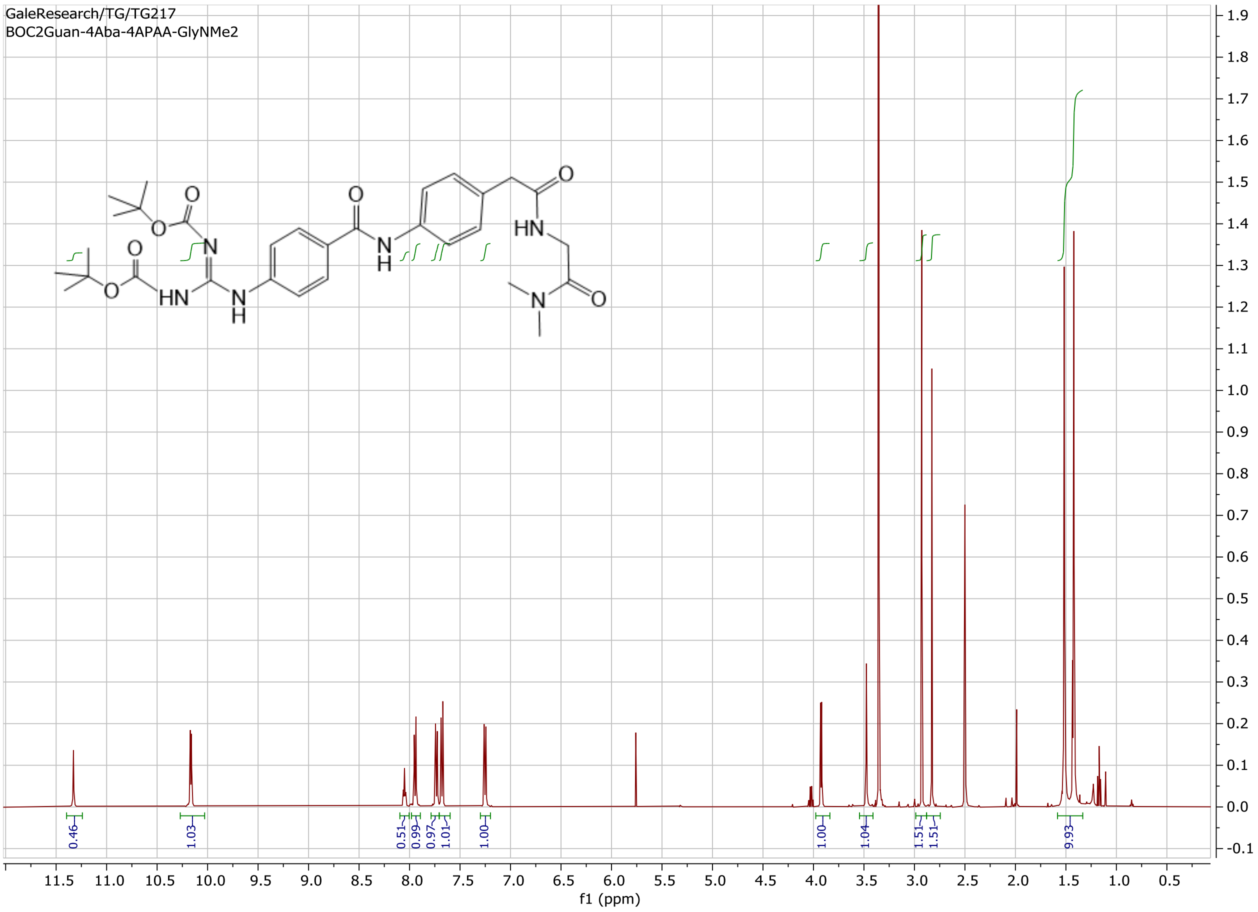

(^{1}LC-MS, reversed phase, H_2O-MeCN)

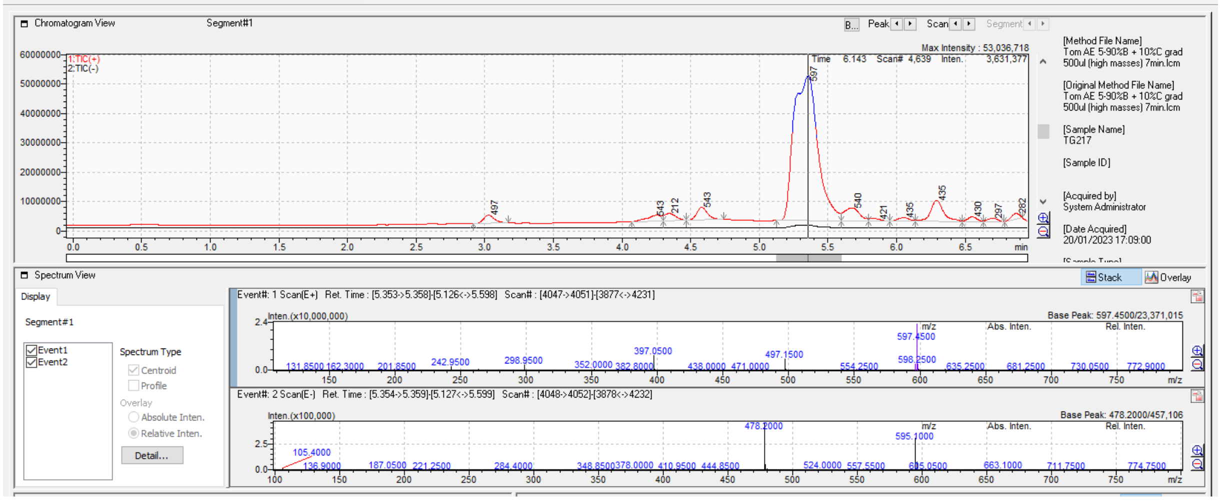

Supplement: Supplementary file 1 [file ijms-26-06761-s001.zip › ijms-3758988-supplementary.pdf]
